# Connecting Calcium-Based Nanomaterials and Cancer: From Diagnosis to Therapy

**DOI:** 10.1007/s40820-022-00894-6

**Published:** 2022-07-18

**Authors:** Shuang Bai, Yulu Lan, Shiying Fu, Hongwei Cheng, Zhixiang Lu, Gang Liu

**Affiliations:** 1 State Key Laboratory of Molecular Vaccinology and Molecular Diagnostics, Center for Molecular Imaging and Translational Medicine, School of Public Health, Xiamen University, Xiamen, 361102 People’s Republic of China; 2 State Key Laboratory of Cellular Stress Biology, Innovation Center for Cell Biology, School of Life Sciences, Xiamen University, Xiamen, 361102 People’s Republic of China

**Keywords:** Calcium-based nanomaterials, Cancer therapy, Calcium signaling, Tumor calcification, Theranostics

## Abstract

As the indispensable second cellular messenger, calcium signaling is involved in the regulation of almost all physiological processes by activating specific target proteins. The importance of calcium ions (Ca^2+^) makes its “Janus nature” strictly regulated by its concentration. Abnormal regulation of calcium signals may cause some diseases; however, artificial regulation of calcium homeostasis in local lesions may also play a therapeutic role. “Calcium overload,” for example, is characterized by excessive enrichment of intracellular Ca^2+^, which irreversibly switches calcium signaling from “positive regulation” to “reverse destruction,” leading to cell death. However, this undesirable death could be defined as “calcicoptosis” to offer a novel approach for cancer treatment. Indeed, Ca^2+^ is involved in various cancer diagnostic and therapeutic events, including calcium overload-induced calcium homeostasis disorder, calcium channels dysregulation, mitochondrial dysfunction, calcium-associated immunoregulation, cell/vascular/tumor calcification, and calcification-mediated CT imaging. In parallel, the development of multifunctional calcium-based nanomaterials (*e.g.*, calcium phosphate, calcium carbonate, calcium peroxide, and hydroxyapatite) is becoming abundantly available. This review will highlight the latest insights of the calcium-based nanomaterials, explain their application, and provide novel perspective. Identifying and characterizing new patterns of calcium-dependent signaling and exploiting the disease element linkage offer additional translational opportunities for cancer theranostics.

## Highlights

- Recent progress of the calcium-based nanomaterials-mediated cancer diagnosis and therapy were summarized.
- Main challenges and clinical translation prospects of calcium-based nanomaterials were discussed.

## Graphical abstract

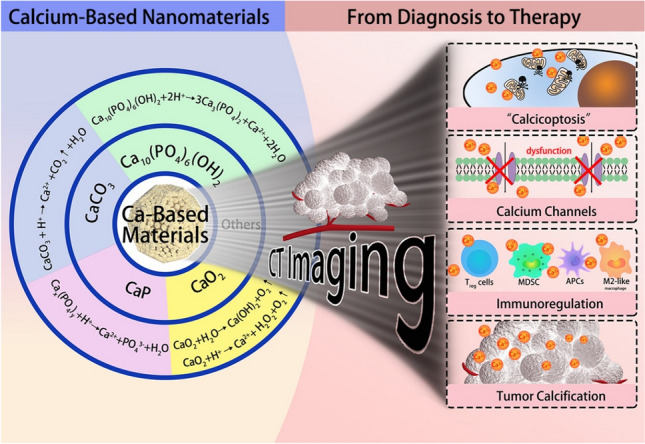

## Introduction

Cell proliferation is one of the most basic life processes and an essential condition for multicellular organisms’ normal development and regulation of [1, 2]. In this case, abnormally elevated levels of cell proliferation and/or deficiencies are the main causes of cell damage, cell senescence, and disease occurrence [3, 4]. The classic example of uncontrolled cell proliferation is cancer [5], in which the survival and development of cancer cells are highly dependent on the availability of sufficient living materials in a series of harsh environments. In this regard, cytoplasmic calcium plays an important role in these processes [6–8]. Calcium ions (Ca^2+^) are the most abundant metal element in the human body and have been identified as a key regulator factor in all cells, which can regulate cells to perform specific biological functions, and are almost involved in every aspect of cellular life, such as cell proliferation, metabolism, migration, death, and gene expression [8, 9]. Over the past decade, there has been a greater understanding of the realignment of calcium signaling in cancer and the role of calcium signaling in critical events [10–12]. Any changes in calcium concentration caused by non-autoregulation of the body will interfere with the normal transmission of calcium signals, thus affecting a variety of normal cellular physiological activities [13].

Recent progress in studying the essential metal elements of the human body has forged a new nexus between disciplines, which has helped to translate basic biochemical research into clinical treatment and diagnosis based on the importance of calcium [14–17]. This transformation is particularly important to cancer because tumor proliferation and invasion are closely related to calcium signals [18]. Calcium signals are finely tuned by complex Ca^2+^ channels, transporters, pumps, exchangers, and calcium-binding proteins [19–22]. Under unstimulated conditions, the cytosolic free Ca^2+^ concentration is maintained at around 100 nM, which is lower than the level of extracellular fluid [23, 24]. There is a 10^4^ ~ 10^5^-fold gradient of Ca^2+^ concentration between outside and inside of cells, providing an opportunity for calcium-mediated tumor therapy [19]. Disruption of intracellular calcium homeostasis can lead to irreversible cell damage mediated by calcium overload, which might be a new potential method for tumor treatment [25, 26]. Additionally, the uncontrolled accumulation of calcium ions in tumor cells can induce abnormal cell signal transduction, raising downstream related events [27]. In recent years, the cell-damaging effects of calcium ions have received increasing attention, and the development of calcium-based nano-therapeutic agents to conduct cell calcium overload and trigger tumor calcification has gradually become a research hotspot [28, 29].

Calcium-based nanomaterials (Ca-NMs) are a direct and effective way to increase the level of cellular basal calcium [29]. Moreover, the inherent non-toxic and biocompatibility of Ca-NMs facilitate their applications in tumor diagnosis and treatment [30]. Therefore, Ca-NMs, such as calcium phosphate (CaP) [31], calcium carbonate (CaCO_3_) [32–34], and hydroxyapatite (HAp) [35, 36], are an attractive class of inorganic calcium-based materials because of their degradation products native presence in the human environment. In addition, calcium peroxide (CaO_2_) is also included in this functional calcium-based material due to its powerful anti-tumor properties. Currently, calcium-related tumor diagnosis and therapeutic mechanisms mainly include the following events: (i) disequilibrium of calcium homeostasis induced by abnormal changes in calcium concentration; (ii) increased intracellular Ca^2+^ induced calcium overload stress, calcium-related cell death, and mitochondrial dysfunction; (iii) intracellular Ca^2+^ imbalance disrupts oxidative phosphorylation and increases ROS production, resulting in oxidative stress-related cell damage; (iv) abnormal calcium levels and their resultant the amplification of oxidative stress co-induced calcium channel dysfunction; (v) calcium ions-related specific immune activation; (vi) the abnormal accumulation of Ca^2+^ for a long time caused tumor calcification, which is conducive to tumor inhibition and improve the poor prognosis; meanwhile, (vii) the specificity enhanced CT imaging induced by calcification is also helpful to diagnosis the therapeutic effect, which showed great significance to realize the clinical theranostics. Based on these personalized diagnostic and therapeutic strategies, it is beneficial to translate calcium-based biochemical studies into the development of calcium-dependent potential clinical therapeutic approaches.

**Fig. 1:**
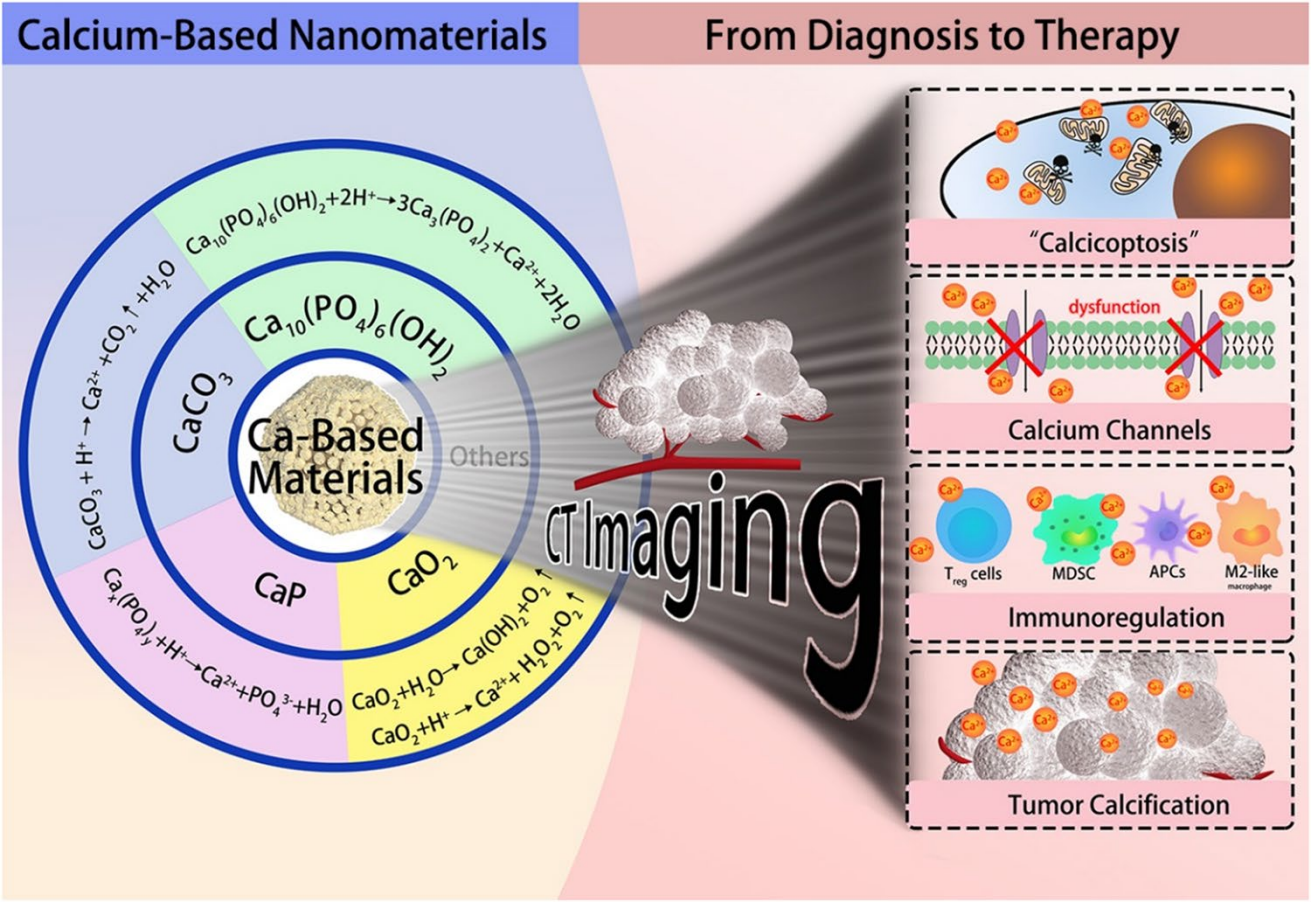
Schematic illustration of calcium-based materials and calcification mediated CT imaging for cancer theranostics. Based on advanced diagnostic and therapeutic strategies, it is beneficial to translate calcium-based biochemical studies into the development of calcium-dependent potential clinical therapeutic approaches and inspire new ideas for the design and development of various calcium-based materials in the future

It is believed that calcium is a ubiquitous major messenger and defects in calcium homeostasis are often closely associated with various pathological changes. A growing body of evidence emphasizes that artificial regulation of cancer calcium is an effective and potential tool in cancer therapy. Various forms of calcium-based nanomaterials have been reported as nanocarriers; diagnostics and therapeutic drugs, and their applications in cancer diagnosis and treatment need to be comprehensively and systematically reviewed in order to better understand their potential value and develop new therapeutic systems. Here, we will summarize the up-to-date progress about the relationship between cancer therapy and the calcium ions, focusing on the medicine delivery, therapies, diagnosis, biosafety, clinical transformation, challenges facing, and application prospects (Fig. 1). We hope such a comprehensive review can help provide important information in cancer diagnosis and treatment to researchers interested in this field and inspire new ideas for the design and development of various Ca-NMs in the future.

## Classification and Physicochemical Properties of Calcium Compounds

In recent years, nanomaterials (NMs) with biocompatibility and biodegradability have become a priority in preclinical research [37, 38]. Using substances or elements contained in the body is the most effective way to solve the problem of biocompatibility. Calcium-based nanocarriers are one of the safest materials, which by-products such as Ca^2+^, PO_4_^3−^, or CO_3_^2−^ are already present in the blood with safe concentrations ranging from 1 to 5 mM [39]. Moreover, most of the calcium-based functional materials possess good tumor microenvironment (TME) response, which can react with H^+^ and thus often employed to design as a pH-sensitive therapeutic system [40, 41]. Given their attractive biocompatibility, long-acting biodegradation, biosafety, and effectiveness, these kinds of calcium salts have broad application prospects in drug delivery and illness treatment. In tumor therapy, the common calcium-based nanomaterials mainly include calcium carbonate (CaCO_3_), calcium phosphate (CaP), calcium peroxide (CaO_2_), and hydroxyapatite (HAp).

### Calcium Carbonate (CaCO_3_)

Calcium carbonate (CaCO_3_) is an important inorganic mineral with a long history of application in different fields. In recent years, CaCO_3_ nanoparticles (NPs) have attracted extensive attention as drug delivery systems (DDSs) in anti-tumor therapy due to their simplicity, low cost, biosafety, biodegradation, and pH sensitivity [42–44]. Such DDSs can directly activate the therapeutic agent to sustained release at the target site. In particular, CaCO_3_ shows great potential in protein/gene delivery and ultrasound (US) imaging-guided therapy [45, 46]. Besides, the functionalization of CaCO_3_ is another aspect of its application potential, which mainly involves providing active targeting, enhancing stability, and improving drug loading capacity [47]. Moreover, the strong buffering function of CaCO_3_ NPs makes it particularly competitive for alleviating acidic TME, which involved in TME reprogramming, and this is one of the factors that most CaCO_3_-based materials can implicate in the regulation of tumor immunity [48, 49]. Most important of all, it is less likely to cause side effects because the degraded products only consist of calcium (which is dominated by the kidneys and deposited in the bones) and carbon dioxide (which is exhaled by the lungs) [45, 50].

**Fig. 2:**
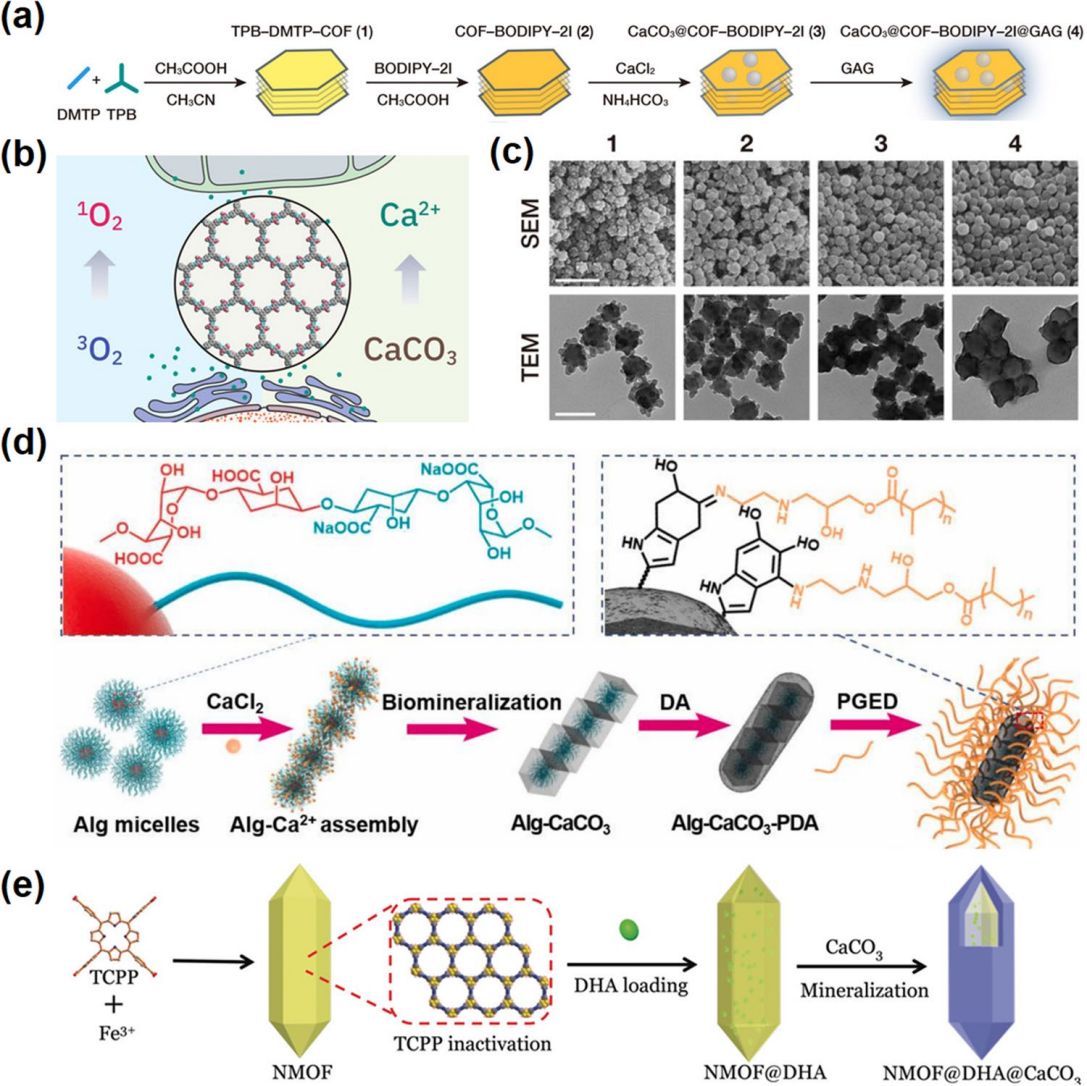
Calcium carbonate-based nanomaterials. **a** Preparation process of CaCO_3_@COF-BODIPY-2I@GAG NMs. **b** Schematic illustration of synergistic induction of intracellular calcium overload by.^1^O_2_ and exogenous calcium delivery. **c** SEM and TEM images of TPB-DMTP-COF, COF-BODIPY-2I, CaCO_3_@COF-BODIPY-2I, and CaCO_3_@COF-BODIPY-2I@GAG [51]. Copyright 2020, Wiley–VCH. **d** Illustration of the synthetic procedure of Alg-CaCO_3_-PDA-PGED (ACDP) gene carriers [54]. Copyright 2021, Elsevier. **e** Schematic illustration of the preparation of the NMOF@DHA@CaCO_3_ nanoplatform [55]. Copyright 2019, Wiley–VCH

As an ideal Ca^2+^ supplier, CaCO_3_ has been skillfully applied in various therapeutic systems. For example, Dong and co-workers used CaCO_3_ as a therapeutic agent during drug delivery and embedded in nanomaterials to prepare CaCO_3_@COF-BODIPY-2I @GAG, in which the covalent organic framework (COF)-based NMs was equipped with TPB-DMTP-COF, photosensitizer (PS)-modified BODIPY-2I, glycosaminoglycan (GAG), and nano-CaCO_3_ (Fig. 2a, c) [51]. The protected nano-CaCO_3_ can be delivered to TME safely without premature leakage and then decomposed in the lysosomes at pH = 5.0, releasing Ca^2+^ synchronously (Fig. 2b). In addition, CaCO_3_ can also be used as a coating material attached to the surface of nanomaterials, which is known as mineralization of the materials [52]. The mineralization methods mainly involve ions spontaneously attached to nanomaterials and then growth in biomimetic mineralization [53]. For instance, Liu et al. conducted gene delivery by mineralizing CaCO_3_ layer on micelles of polysaccharide sodium alginate with Ca^2+^ as growth points to prepare Alg-CaCO_3_ [54]. Subsequently, polydopamine (PDA) and polyethylene glycol (PEG) modifications were implemented to enhance the photothermal effect and biocompatibility (Fig. 2d). Wan et al. constructed a synergistic therapeutic strategy by Fe-based MOF coated with CaCO_3_ to prevent leakage of the loaded drug dihydroartemisinin (DHA), resulting in a triple-treatment consisting of DHA-Fe^2+^-mediated chemodynamic therapy (CDT), photosensitive MOF-mediated photodynamic therapy (PDT), and Ca^2+^-mediated calcium overload (Fig. 2e) [55]. Moreover, as a commonly used food/pharmaceutical excipient [56], CaCO_3_ is also used in anti-tumor sprays. Gu et al. prepared a sprayed bio-reactive immunotherapeutic fibrin gel with the function of inhibiting local tumor recurrence and distant development [57]. Biocompatible CaCO_3_ is incorporated into fibrin gels as a release reservoir for immunomodulators, as well as regulating proton balance in the tumor environment.

**Fig. 3:**
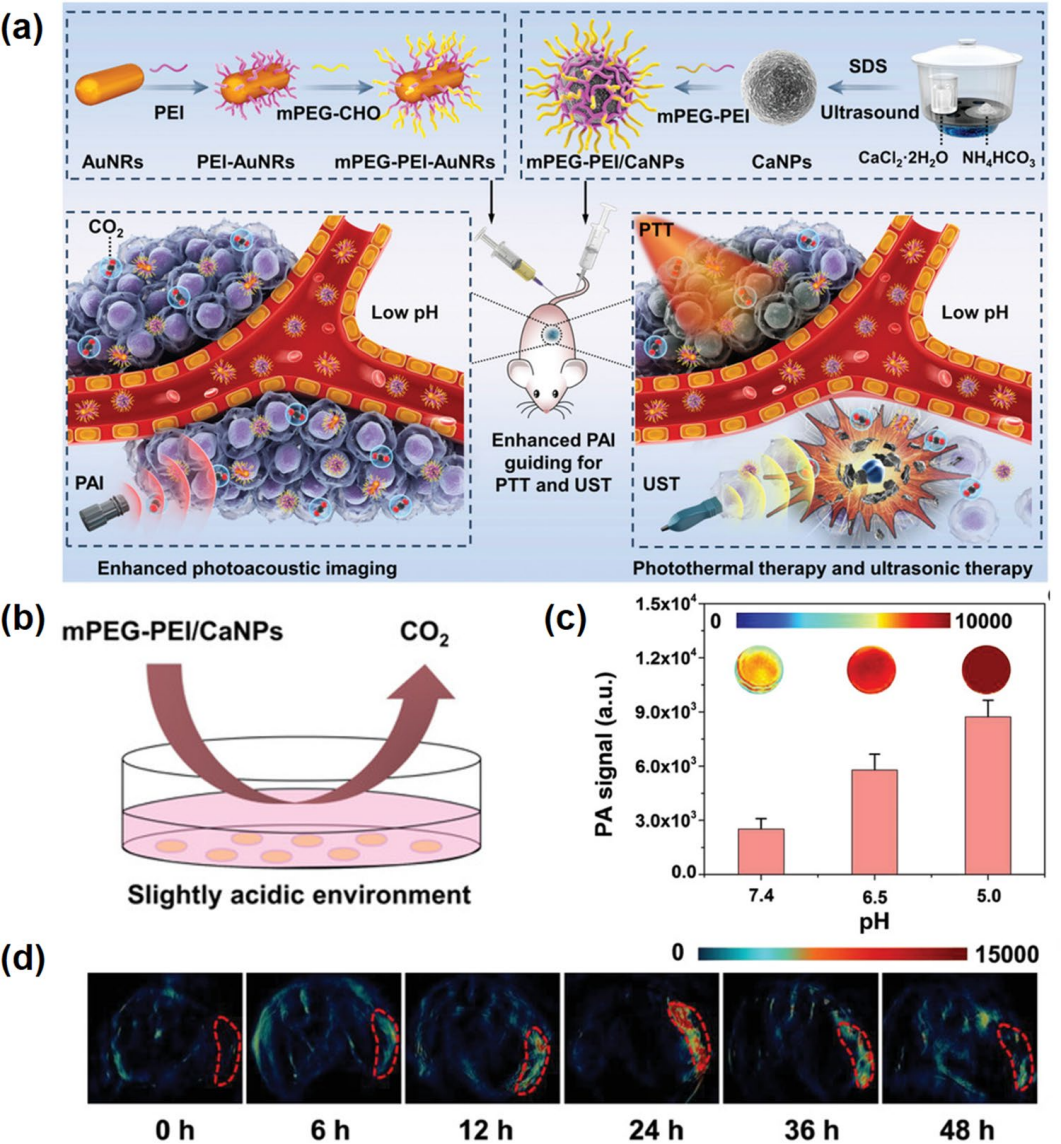
Calcium carbonate-enhanced ultrasonic imaging. **a** Schematic diagram of synthesized mPEG–PEI–AuNRs and mPEG–PEI/CaNPs with enhanced PA imaging for the synergistic of photothermal therapy and ultrasonic therapy. **b** Schematic diagram of CO_2_ bubbles produced by mPEG-PEI/CaNPs in a simulated acidic TME. **c** PA signals of mPEG-PEI-AuNRs and mPEG-PEI/CaNPs under different pH values. **d** PA signals of mPEG-PEI-AuNRs and mPEG-PEI/CaNPs in tumor tissues [50]. Copyright 2021, Wiley–VCH

On the other hand, CaCO_3_ can also react with protons to produce carbon dioxide (CO_2_) gas with the form of bubbles. These bubbles can be served as a sensitive and biocompatible contrast agent applied in ultrasonic diagnostic imaging because microbubbles can improve the contrast of ultrasonic imaging on account of their special acoustic effect [45, 58]. For example, Chen's group reported a bubble-producing CaCO_3_ NMs for enhancing bio-imaging (Fig. 3a) [50]. These bubble-producing mineralized NPs can generate CO_2_ at the acidic pH value of the tumor, and the photoacoustic (PA) signal is enhanced with the formation of bubbles, which can monitor the concentration of drugs at the lesion site and guide precise tumor treatment (Fig. 3b–d). Meanwhile, the produced CO_2_ bubbles can burst by ultrasound irradiation in a moment, inducing cell death and suppress tumor develop. This strategy of combining of diagnosis and therapeutic will has broad application prospects in eradicating tumors.

### Calcium Phosphate (CaP)

In the normal course of life, the human body can spontaneously form calcium phosphate (CaP)-based composites (e.g., bones and teeth) through biological mineralization [59, 60]. As a major inorganic composition of hard bio-tissue, CaP has basic biocompatibility and biodegradability, which had been approved by the Food and Drug Administration (FDA) as a biomaterial. In addition, CaP holds similar pH sensitivity with CaCO_3_, which is stable at neutral pH while decomposition under acidic conditions produces Ca^2+^ and PO_4_^3−^, making it a good nanomaterial for pH-responsive DDSs [39]. They are often used in combination with lipids to enhance cellular internalization [61]. Moreover, CaP can effectively avoid immune rejection and improve bioavailability due to its widespread presence in organism [59, 62].

Inspired by biological mineralization, CaP-based multifunctional nanocarriers have played an excellent role in drug delivery, cancer diagnosis, and treatment, demonstrating high potential for cancer theranostics. As CaP is the main component of human bone, which can specifically promote the application of these nanocarriers in the fields of bone tumors and bone defects [63, 64]. Simultaneously, CaP is also the main mineral form of tissue calcification, which is effective in enhancing tumor necrosis and improving prognosis [59]. Overall, CaP has undoubtedly contributed to significant advances in cancer therapeutics, from tissue engineering to drug delivery. Because of its good biochemical properties and interlocking biological effects in vivo, it is considered one of the most promising calcium-based materials.

**Fig. 4:**
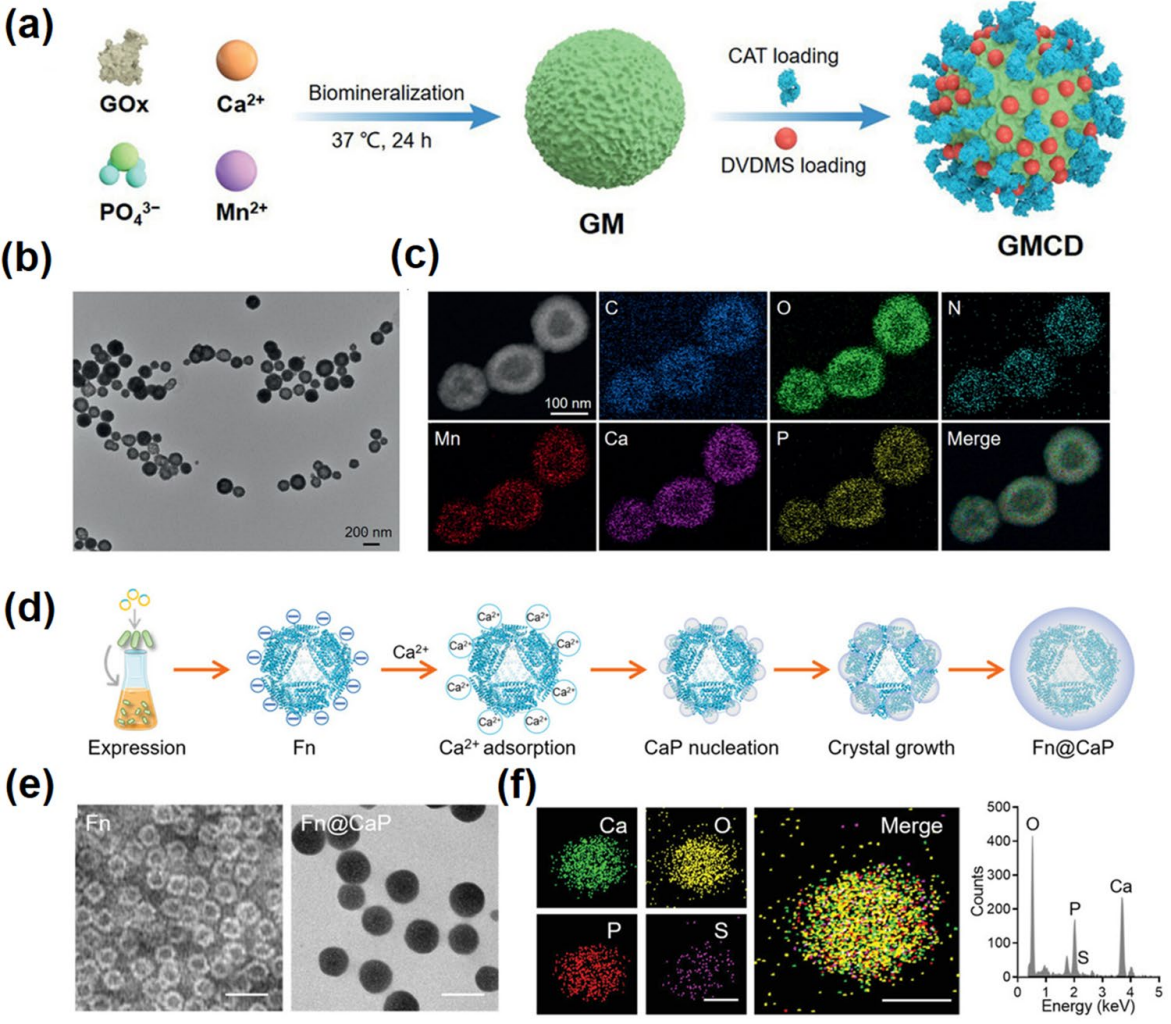
Calcium phosphate-based nanomaterials. **a** Synthetic procedure of GMCD. **b** The TEM images of GM. **c** Elemental mapping of GM [68]. Copyright 2021, Wiley–VCH. **d** Synthetic process of the Fn@CaP nanoplatform. **e** TEM images of Fn and Fn@CaP. **f** Elemental mapping analysis (left panel) and energy dispersive spectroscopy (EDS) analysis (right panel) of Fn@CaP [70]. Copyright 2022, Wiley–VCH

CaP was first studied in the 1970s as a gene vector with good biocompatibility for gene transfection [65]. Since then, CaP has been expanded and developed as a bioactive agent for diagnosis, imaging and cancer therapy [66]. It must be mentioned that CaP does improve the precision treatment of cancer efficacy, showing great potential for clinical application [67]. CaP is mainly involved in cancer therapy through the following ways, including as a carrier and as a mineralized coating. When utilized as nanocarriers, these materials show excellent biocompatibility and have selectively toxic at comparable concentrations even without drugs, making them friendly for cancer treatment. Huang’s group built a CaP-based oxygen self-supplied nanosystem for alleviating tumor hypoxia and activates tumor-specific cascade catalysis [68]. This nanoplatform is composed by co-loading catalase and photosensitizer DVDMS in the mixed CaP-based nanoparticles, in which calcium phosphide acts as a carrier to provide drug delivery site and plays the corresponding release function (Fig. 4a–c).

Biomineralization is one of the most common modification methods of CaP. Tan et al. reported a core–shell nanosonosensitizer agent (TiO_2_@CaP) that activates reactive oxygen species (ROS) production and dissolves its capsid in acidic TME under ultrasonically activated, which can change the state of ROS generation from an “OFF” to an “ON” state and simultaneity release excess Ca^2+^ to induce oxidative stress [69]. In addition, CaP bio-mineralization can effectively improve the stability of protein, peptide, enzyme, and other biological macromolecules under physiological conditions, maintain their biological activity, and achieve long-term sustainable drug release. For example, Wang and co-workers coated ferritin (Fn) with CaP to enclose Fn within a “temporary protective shell” by biomineralization technology to increase the enrichment of Fn in the target region, and the TME-responsive dissolution of CaP shell can not only neutralize tumor acidity but also induce intratumor immune regulation and tumor calcification (Fig. 4d–f) [70]. Due to its well-behaved biocompatibility, biodegradability, and controlled release behavior, CaP nanoplatform provides a profound paradigm for effective cancer therapy and has great potential for in clinical transformation.

### Calcium Peroxide (CaO_2_)

Calcium peroxide (CaO_2_) has been widely used in tumor therapy because it can provide hydrogen peroxide (H_2_O_2_) and oxygen (O_2_) in TME without external stimulation and simultaneously release a large number of free calcium ions rapidly, promoting cell death through oxidative stress enhancement induced by calcium overload and H_2_O_2_ [71–75]. It is gratifying that the CaO_2_-mediated cell killing effect is not limited to specific tumor type, which could against a variety of tumor types including liver cancer, colon cancer, lung cancer, and breast cancer, whereas normal cells are protected from harm because they are more resistant to adverse effects than tumor cells [76, 77]. In addition, CaO_2_ is hydrolyzed at an accelerated rate in the acidic TME, resulting in instantaneous intracellular calcium accumulation and amplification of oxidative stress. The acid sensitivity of CaO_2_ is mainly because acidic conditions accelerate the proton generation process of water ionization. The decomposition rate of calcium peroxide is related to the concentration of surrounding protons, and the product is Ca^2+^ and H_2_O_2_. Here, the production of H_2_O_2_ will cause acute oxidative stress in cells, resulting in abnormal intracellular calcium channel function, which prevents excessive calcium ions pumped out of cells and makes intracellular Ca^2+^ concentration difficult to adjust to the normal values, ultimately leading to calcium overload mediated cell necrosis [28]. Moreover, for tumor cells with severely downregulated CAT, transient enhanced oxidative stress will promote protein damage, leading to abnormal function of calcium ion channels, which in turn cause of irreconcilable abnormal accumulation of intracellular calcium. This unique biological function severely obstructs the steady delivery of calcium messages and induces cell death.

**Fig. 5:**
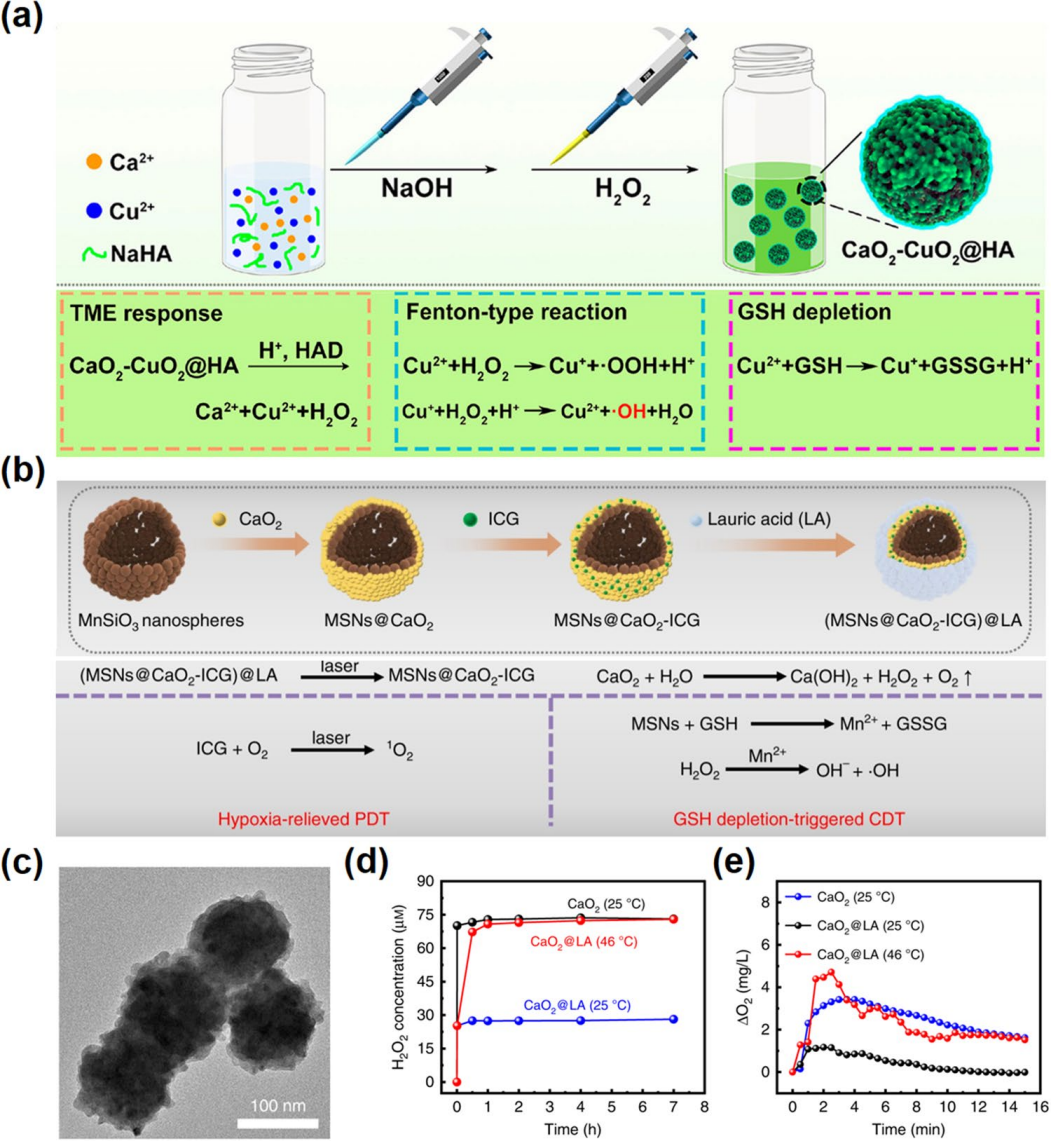
Calcium peroxide-based nanomaterials. **a** Synthesis procedure (top panel) and antitumor performance (low panel) of CaO_2_ − CuO_2_@HA NPs [72]. Copyright 2022, American Chemical Society. **b** Schematic illustration of the synthesis (top panel) and therapeutic mechanism (low panel) of (MSNs@CaO_2_-ICG) @LA NPs. **c** TEM images of MSNs@CaO_2_. **d** H_2_O_2_ cumulative release profile and **e** O_2_ generation in PBS [79]. Copyright 2022, Springer Nature

However, due to the severe water instability of CaO_2_, it will lead to leakage of H_2_O_2_ and Ca^2+^ in blood circulation, causing the unfavored side effects to the body, and this bottleneck limits its applicability. Therefore, most of the current treatments based on CaO_2_ are aimed at reducing its non-specific toxicity and improving its stability in blood circulation [28, 72, 78, 79]. In this regard, great research results have been presented; for instance, Liu and co-workers reported a hyaluronic acid (HA)-modified CaO_2_ and CuO_2_ nanocomposite to delay the hydrolysis of peroxide in a normal physiological environment and simultaneously realize synergistic antitumor effect by a combination of multiple metal peroxides [72]. Sodium hyaluronate-modified nano-CaO_2_ are relatively stable in the bloodstream until they reach the TME, where the protective covering is degraded by TME-overexpressed hyaluronidase to release the drug. After effective accumulation at the TME, plenty of H_2_O_2_ was released in TME, and then, Fenton reaction between released Cu^2+^ and generated H_2_O_2_ further produced a large number of hydroxyl radicals, which enhanced the amplification of transient oxidative stress in cells (Fig. 5a). In addition to using TME signals to stimulate cleavage of the cladding material, external stimuli can also act as a response switch. For instance, Liu et al. designed an calcium-based nanoparticle of (MSNs@CaO_2_-ICG)@LA with H_2_O_2_/O_2_ self-supplied, which is composed of manganese silicate (MSN)-loaded nano-CaO_2_ and indocyanine green (ICG) and further modified with lauric acid (LA, phase change point: 44 ~ 46 °C) on the surface (Fig. 5b–c) [79]. Under the irradiation of 808 nm laser, ICG can produce singlet oxygen (^1^O_2_) and simultaneously create a high temperature to melt LA and release CaO_2_. Here, the authors focused on the synergistic ROS generation of calcium peroxide system utilizing opening source and reducing expenditure, in which H_2_O_2_ produced by CaO_2_, H_2_O_2_-mediated CDT, and oxygen-mediated PDT jointly constitute the open-source strategy, and the consumption of GSH induced by MSN protects reactive oxygen species from being cleared is a reduce-consumption strategy (Fig. 5d–e). This drug delivery strategy has shown excellent tumor-suppressive effects in vivo and in vitro, which improves cancer treatment approach based on ROS from multiple aspects. CaO_2_ is a kind of milestone existence in the calcium-based material development in tumor therapy, which can not only combine the dual functions of Ca^2+^ and H_2_O_2_ to induce cell apoptosis, but also provide important guiding significance for the development of “green tumor therapy.”

### Hydroxyapatite (HAp)

Another calcium-based material commonly used in cancer therapy is hydroxyapatite (HAp, Ca_10_(PO_4_)_6_(OH)_2_). HAp is the main inorganic mineral in human bones and teeth, which has stronger mechanical and functional properties [80, 81]. Their development in tissue-engineered scaffolds and as a matrix for bones and teeth are already ripe, and beyond that, they have been exploited for systemic therapeutics as bioactive carriers in the field of cancer therapy, especially in the treatment of malignant bone neoplasms [82, 83].

**Fig. 6:**
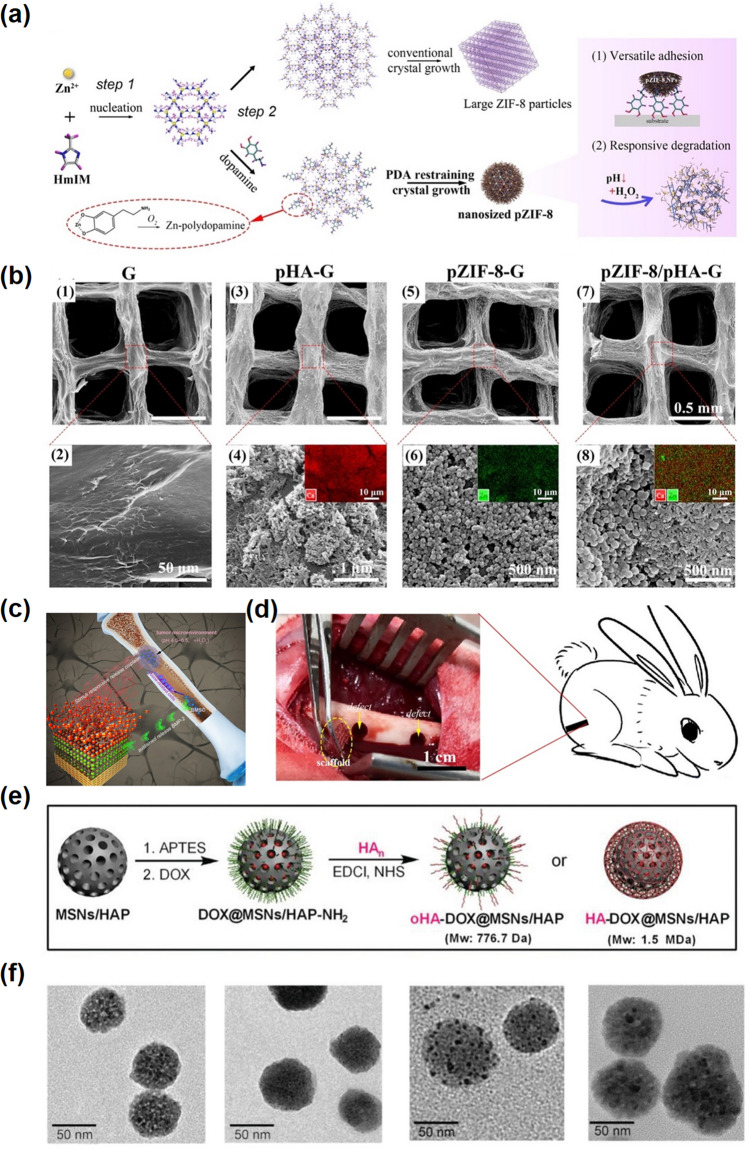
Hydroxyapatite-based nanomaterials. **a** Scheme of the fabrication process of polydopamine-hybrid ZIF-8 nano-MOF. **b** Microstructure of different bone-filled scaffolds. The photographs inserted in (4), (6), and (8) are the EDS images. **c** The scaffold had the dual function of antitumor and bone regeneration, which can release cisplatin and BMP-2 in acidic TME to inhibit tumor growth and continuously achieve bone regeneration. **d** Scaffold implantation of femur condyle defect model in the rabbit [86]. Copyright 2021, Elsevier. **e** Synthetic process of oHA-DOX@MSNs/HAP NPs or HA-DOX@MSNs/HAP NPs. **f** TEM images of MSNs/HAP NPs, DOX@MSNs/HAP NPs, oHA-DOX@MSNs/HAP NPs, and HA-DOX@MSNs/HAP NPs, respectively [87]. Copyright 2019, Wiley–VCH

Cancer-related bone defect is the main cause of failure in clinical treatment of bone neoplasms [84]. Therefore, it is urgent to design function bone filling materials with both bone tissue regeneration and anti-tumor properties [85]. From the perspective of bone filling, an optimal bone scaffold should have the following basic characteristics: biocompatibility, mechanical strength, and interconnected porous system. For instance, Jiang et al*.* presented a bone-filled scaffold prepared by 3D printing, which can simultaneously load therapeutic drugs and bone regeneration factors for the treatment of bone neoplasms [86]. Such a bone filler template was incorporated by alternating assembly of polydopamine-hybrid ZIF-8 and PDA-decorated HAP on the gelatin-based scaffold surface layer by layer (Fig. 6a–b). As a carrier platform, the scaffold was loaded with bone regeneration factor (BMP-2) and cisplatin, which could induce osteogenic differentiation and inhibit tumor growth well (Fig. 6c–d). In addition, HAp is sensitive to pH and degrades easily under weak tumor acidic TME (6.5–6.8), making it an excellent drug delivery vehicle. Kang and his colleagues used Hap-doped mesoporous silica nanoparticles (MSN) as a carrier; it can not only achieve pH-responsive drug release but also improve drug loading rate and excellent therapeutic efficacy (Fig. 6e–f) [87].

Hydroxyapatite has also been fully investigated and applied in clinical level. For example, Cornell et al. reported an alternative autologous bone graft substitute named Collagraft® that contained hydroxyapatite of 65% by mass. Their prospective clinical trial showed that the product achieved the same efficacy as autologous cancellous grafts in the treatment of acute long bone fractures [88]. The product has already received FDA approval in 1994. Meanwhile, hydroxyapatite has certain clinical potential in repairing bone defect of bone tumor. For instance, Liu et al. developed a novel height-adjustable vertebral body (AHVB) prosthesis modified with the nano-hydroxyapatite/polyamide-66 (n-HA/PA66) for clinical reconstruction of thoracolumbar structural stability after spinal tumor resection. This novel prosthesis was performed on 7 patients with thoracolumbar spinal tumor resection, showing a favorable prospect for clinical application [89].

### Other Forms of Ca-NMs

**Fig. 7:**
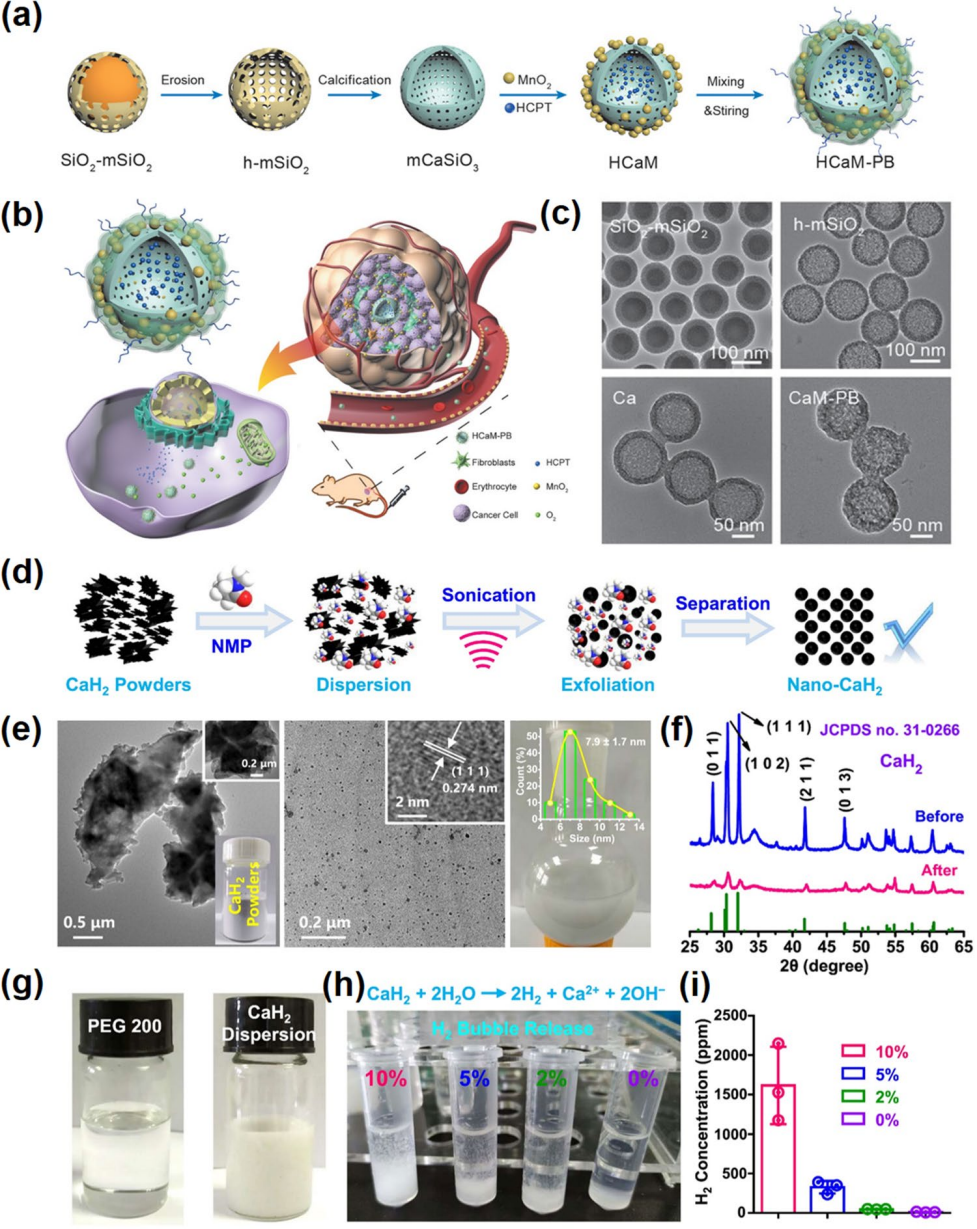
Other forms of Ca-NMs. **a** Synthesis process of HCaM-PB. **b** Schematic diagram of HCaM-PB application in tumor therapy. **c** TEM images of SiO_2_@mSiO_2_ NPs, h-mSiO_2_ NPs, Ca NPs, and CaM-PB NPs [95]. Copyright 2019, Wiley–VCH. **d** Synthesis procedure of nano-CaH_2_. **e** TEM images of the CaH_2_ powders (left panel) and the nano-CaH_2_ (middle panel). A photograph of commercial CaH_2_ powders is inserted in the left panel and a lattice structure of nano-CaH_2_ is inserted in the middle panel. A photograph of nano-CaH_2_ in N-methylpyrrolidone (right panel). The inserted figure shows the hydration size of nano-CaH_2_. **f** XRD of nano-CaH_2_ and CaH_2_ powders. **g** A photograph of PEG-200 (left panel) and nano-CaH_2_ dispersed in PEG-200 solvent (right panel). **h** The photograph of the H_2_ produced in different proportions of H_2_O solution. **i** The generated H_2_ concentration in different solutions determined by gas chromatography [92]. Copyright 2022, Elsevier

Other calcium-based biomaterials such as calcium silicate (CaSi) [90], calcium fluoride (CaF_2_) [91], and calcium hydride (CaH_2_) [92] have also been studied. CaSi biomaterials such as CaSiO_3_ and Ca_2_SiO_4_ are widely used in bone tissue engineering served as bioglass, bioceramics, and bone cement [93, 94]. Nanostructured CaSi has a porous structure and high specific surface area with pH-responsive degradation ability [95]. In addition, Ca^2+^ can be used as the inherent active site to anchor the -COOH or -OH groups of drug molecules to improve the load rate. Thus, it often served as an ideal carrier system to support drug accumulation in tumors without premature leakage into the blood circulation. For example, Guo and his colleagues synthesized MnO_2_-loaded mesoporous CaSiO_3_ nanoparticles with bovine serum albumin (BSA) and PEG co-modified to act as a nanoplatform with relieving hypoxic and therapeutic effect (Fig. 7a–c) [95]. After decomposition of hypoxia-related MnO_2_, mesoporous CaSiO_3_ nanopore size gradually increased in the acidic tumor microenvironment, leading to continuous drug release. Due to the presence of mesoporous CaSiO_3_ nanoparticles, the synthetic material achieves a sequential therapeutic function, and the release mode enables higher drug concentrations after improved hypoxia.

Calcium fluoride (CaF_2_) has good biocompatibility and is mainly used as a promoting agent for tooth or bone reconstruction [96]. More importantly, CaF_2_ is a special up/down-conversion luminescent matrix material, which can play a unique role in fluorescence imaging (FLI). For instance, Dong et al. reported that CaF_2_:Tm^3+^, Yb^3+^ nanoparticles can achieve a tissue penetration depth of 2 mm and have high two-photon excited fluorescence efficiency, which was candidate for in vivo FLI [97]. In addition, CaF_2_ can also act as a scintillator with strong X-ray-material interactions to assist tumor radiotherapy. Wang et al. first found that CaF_2_ NPs may be selectively toxic to tumor cells. They developed a europium (Eu)-doped CaF_2_ NPs to inhibit the growth and migration of osteosarcoma [98]. Moreover, CaF_2_ material can increase cell adhesion and inhibit tumor metastasis and cooperate with Eu to inhibit the recurrence and metastasis of residual tumor cells. Additionally, CaF_2_ also plays a corresponding role in photodynamic therapy (PDT) [99]. Due to the strong afterglow emission with a long lifetime of CaF_2_: Tm nanoparticles, thus PpIX sensitizer and CaF_2_: Tm nanoparticles conjugation can be used as energy sources to improve PDT. Further, CaF_2_-based nanoparticles can be developed as a platform for multimodal imaging-guided therapy [100].

Calcium hydroxide (CaH_2_) is a common inorganic material for hydrogen storage, which has been found to have a good tumor inhibition effect in recent years [92, 101]. CaH_2_ can react with H_2_O to produce Ca^2+^, hydrogen gas (H_2_), and hydroxyl (OH^−^), in which Ca^2+^ can induce calcium overload, OH^−^ can neutralize acidic TME, and the high validity of H_2_ can achieve hydrogen therapy. For example, Liu et al. prepared CaH_2_ nanoparticles for antitumor therapy by liquid-phase exfoliation (Fig. 7d–f) [92]. They dispersed CaH_2_ into low-molecular-weight polyethylene glycol to protect it from direct contact with water (Fig. 7g). As shown in Fig. 7 h–i, the nano-CaH_2_ dispersion in different proportions of water and ethanol solutions was able to observe the release of H_2_. In addition, it was proved that nano-CaH_2_ could induce apoptosis of colon and breast cancer cells in vitro, which was mainly due to intracellular calcium overload and H_2_ production inhibiting cell function and eventually leading to cell death. Moreover, they explored the value of nano-CaH_2_ in vivo interventional embolization therapy, which can relieve hypoxia and metastasis of tumors caused by embolism. They dispersed nano-CaH_2_ into lipiodol and introduce them into in situ rabbit model of hepatocellular carcinoma. The introduced nano-CaH_2_ has huge potential to break the limitations of interventional transarterial embolization (TAE) and improve the therapeutic effect of liver cancer.

## Calcium-Based Nanomaterials for Cancer Diagnosis and Therapy

Calcium ions are significantly important in many cellular processes; abnormal intracellular calcium ions can disrupt calcium homeostasis and influence cellular machinery [18]. Calcium ions can damage mitochondrial function, thereby enhancing oxidative stress in cancer cells [102–105]. In addition, dysregulation of calcium channels resulting by abnormal oxidative stress can further aggravate tumor necrosis, which is often defined as calcium interference therapy. Meanwhile, tumor calcification can specifically enahnce CT imaging, which is help to accelerate the progress of the integration of clinical diagnosis and therapy [106–108]. In addition, calcium can also regulate the tumor immune microenvironment, playing the uniqueness of calcium ions in tumor immunotherapy [109]. Exploring the important role of Ca^2+^ in cancer therapy is helpful to thoroughly understand the calcium–cancer mechanism and accelerate the application of calcium in cancer therapy.

### Ion Interference Therapy

In recent years, studies based on biodegradable nanomaterials have shown that the inherent anti-cancer activity can be regulated by disturbing the intracellular ion balance due to the related biological activity of their degradation products, which is known as ion interference therapy (IIT) [110, 111]. Of these aspects, iron-based materials (“ferroptosis”) [112–114] and sodium chloride [115, 116] provided the good demonstration. Therefore, ion-interfering cancer therapy is expected to become a new therapeutic tool as a supplement to traditional clinical cancer therapy. As a widespread intracellular metal ion, Ca^2+^ homeostasis is important for normal life processes; thus, regulating calcium concentration to mediate calcium interference therapy has broad research prospects. The ideal approach is to promote the cancer cells collect the inherent Ca^2+^ on their own, specifically attacking and destroying themselves in a highly efficient and side effect-free manner. However, the maintenance mechanisms of intracellular Ca^2+^ levels, such as transmembrane transport and subcellular organelle buffering, are highly autonomous processes, making it difficult to regulate calcium lethal concentrations by intracellular basal calcium level [117]. Therefore, Ca-NMs are the most promising strategy to increase intracellular calcium levels directly and induce calcium ion interference therapy.

**Fig. 8:**
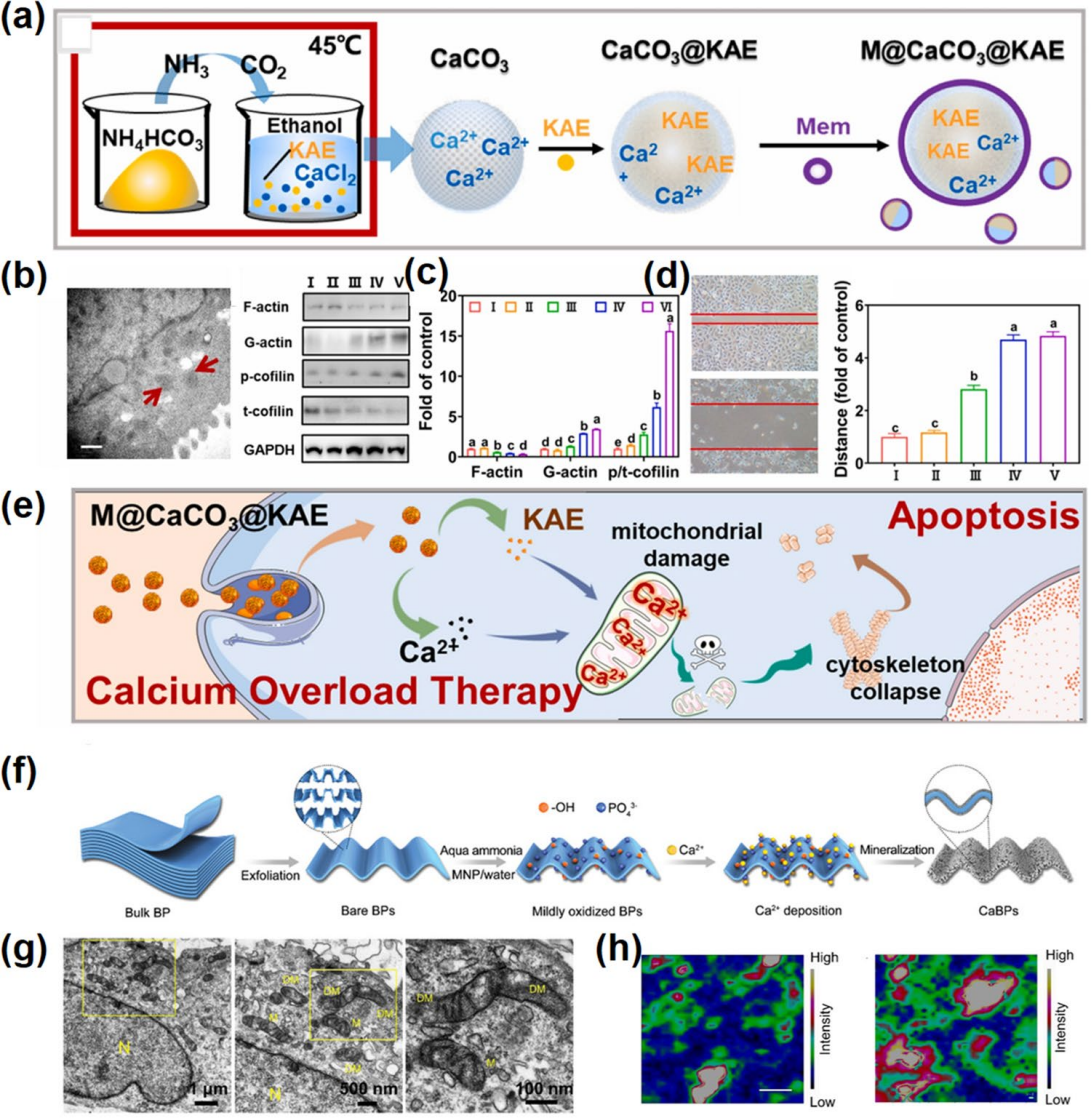
Ca-NMs mediated ion interference therapy. **a** Illustration of the preparation process of M@CaCO_3_@KAE NPs. **b** TEM image of mitochondria after M@CaCO_3_@KAE NPs treatment. **c** Protein levels (left panel) and the intensity (right panel) of A549 cells. **d** Migration ability (left panel) and the quantitative results of migration distance (right panel) of A549 cells. **e** Schematic diagram of M@CaCO_3_@KAE NP-mediated apoptosis [42]. Copyright 2021, Elsevier. **f** Synthetic process of CaBPs. **g** Bio-TEM images of in CaBPs treatment group, where N represents the nucleus, M represents the healthy mitochondria, and DM represents the damaged mitochondria. **h** Raman mapping of A ^1^_ g_ Raman peak characteristic intensity based on BPs in isolated mitochondria from CaBPs-treated (left panel) and unmodified BPs-treated (right panel) MCF-7 cells [119]. Copyright 2020, Wiley–VCH

One of the most relevant results of ion interference therapy induction is mitochondrial damage caused by calcium overload [104]. For example, Chu et al. reported a combined therapy strategy to achieve calcium overload amplification efficiency [42]. The Kaempferol-3-O-rutinoside (KAE) was loaded into CaCO_3_ NPs and coated with the tumor cell membrane (M), in which CaCO_3_ served as a calcium ions donor and KAE as a calcium regulator can destroy normal regulation of calcium homeostasis and promote calcium influx (Fig. 8a). Upon arrival at the tumor, M@CaCO_3_@KAE responded specifically to the TME, releasing KAE and Ca^2+^. Then, KAE effectively breaks the calcium homeostasis, and Ca^2+^ significantly aggravates and amplifies ion-interfering mediated calcium overload. In the meantime, the structure and function of mitochondrial are disrupted, resulting in cell architecture breakdown and oxidative stress damage, which hinders cell proliferation, migration, and invasion, and eventually leads to cell apoptosis (Fig. 8b–d).

In addition to killing cells by their physicochemical properties, ions changing the osmotic pressure is another form of ion interference therapy [115, 116, 118]. The ion gradient inside and outside the cell is crucial for the normal function of the cell. The passive transport driven by concentration gradient regulates basic cell life processes, including nutrient transport, ion transport, and pH regulation. Any manipulation that alters the osmotic pressure of the cell will cause the destruction of the cytoskeleton [115, 116]. For example, NaCl NPs bypass the ion transport passage through endocytosis function enter to the tumor cells and quickly dissolve in tumor cells to cause a surge of osmotic pressure to make cell lysis [116]. This treatment is not limited by the type of cancer, which showed obvious cytotoxicity in several types of cancer cells but little cytotoxicity in normal cells due to the low initial Na^+^ level. Similarly, Pan et al. reported a in situ CaP mineralization strategy based on black phosphorus (BP) to induce ion-mediated osmotic imbalance (Fig. 8f) [119]. Moreover, by using BP as a synthesis template and phosphate source, the synthetic CaP mineralized BP(CaBPs) not only retain the inherent biochemical activity but also showed a high loading capacity for various drug/fluorescent dye molecules, thus achieving effective biological imaging and tracking. In the slightly acidic TME, the degradation and release of CaBPs can lead to the sharp increase in intracellular Ca^2+^ and PO_4_^3−^, resulting in the change of intracellular ion osmotic pressure. Mitochondrial membrane potential (MMP) is highly sensitive to changes in intracellular ion osmotic pressure, and as one of the most susceptible target organelles, mitochondrial function is severely damaged (Fig. 8g). The characteristic A^1^_g_ peak of CaBPs could be detected in isolated mitochondria, indicating that a large number of CaBPs existed in mitochondria are enough to cause changes in osmotic pressure and cause mitochondria-mediated apoptotic cell death (Fig. 8h). This new approach has great promise in cancer treatment, and it is believed that with the further exploration and utilization of the tumor microenvironment, “ion interference therapy” will definitely provide benefits for the development of anti-cancer therapy.

### Calcium Channels and Calcium Pumps

Complex calcium level changes controllers in cells are the main tools for regulating calcium homeostasis-calcium channels and pumps [120–122]. Nowadays, more and more evidence indicates that calcium channels or calcium pump is involved in tumorigenesis and progression [10]. In fact, altered expression of Ca^2+^-transporting molecules can promote tumor cell growth, para-tumor angiogenesis, uncontrolled proliferation, and metastasis. Calcium channels mainly fall into two major categories, voltage-gated calcium channel (VGCC) and non-voltage-gated calcium channel (NVGCC). There are five types of VGCC, including L-type/ P-type/ N-type/ R-type /T-type calcium channels [123]. NVGCC mainly includes ligand-gated channels (LGCs), receptor-operated channels (ROCs), store-operated calcium channels (SOCs), and transient receptor potential (TRP) family channels [124]. There is found that the development of cancer is often accompanied by significant remodeling in the corresponding proteins of Ca^2+^ channels. For instance, T-type Ca^2+^ channels are up-regulated in melanoma [125], TRP family channels in breast cancer patients were up-regulated [126], Ca^2+^ release-activated Ca^2+^ (CRAC) channel is highly expressed in colorectal cancer cells [127], and etc. Besides, a cohort study of 295 breast cancer patients showed that the protein expression level of STIM1 (SOCs-associated protein) was positively correlated with tumor growth, and the higher STIM1 mRNA levels, the shorter the survival of patients [128].

As a matter of fact, some calcium transporters are not only regulators of carcinogenic signals, but their altered expression is also a promoter of some cancers. For example, epithelial–mesenchymal transition (EMT) is closely linked to the progression of metastasis and recurrence of cancer, which controls the malignant phenotype of almost all cancer cells [129]. An increasing number of researches have suggested that calcium channels in plasma membrane and endoplasmic reticulum are involved in the process of EMT in different cancers [130]. For example, CRAC channels, TRP channels, and VGCCs are implicated in the regulation of EMT in breast cancer [131]. In terms of tumor suppression, calcium channels are involved in the apoptosis of multi-drug resistance resisting cells. It was found that the loss of CACNA1C expression positively correlated with rituximab-mediated immunochemotherapy resistance in diffuse large B-cell lymphoma [132]. In addition, elevated orai3 has been reported to promote chemo-resistance through the P53 mechanism [133]. Anyway, the change of calcium channel expression is inextricably linked with the occurrence and development of cancer.

**Fig. 9:**
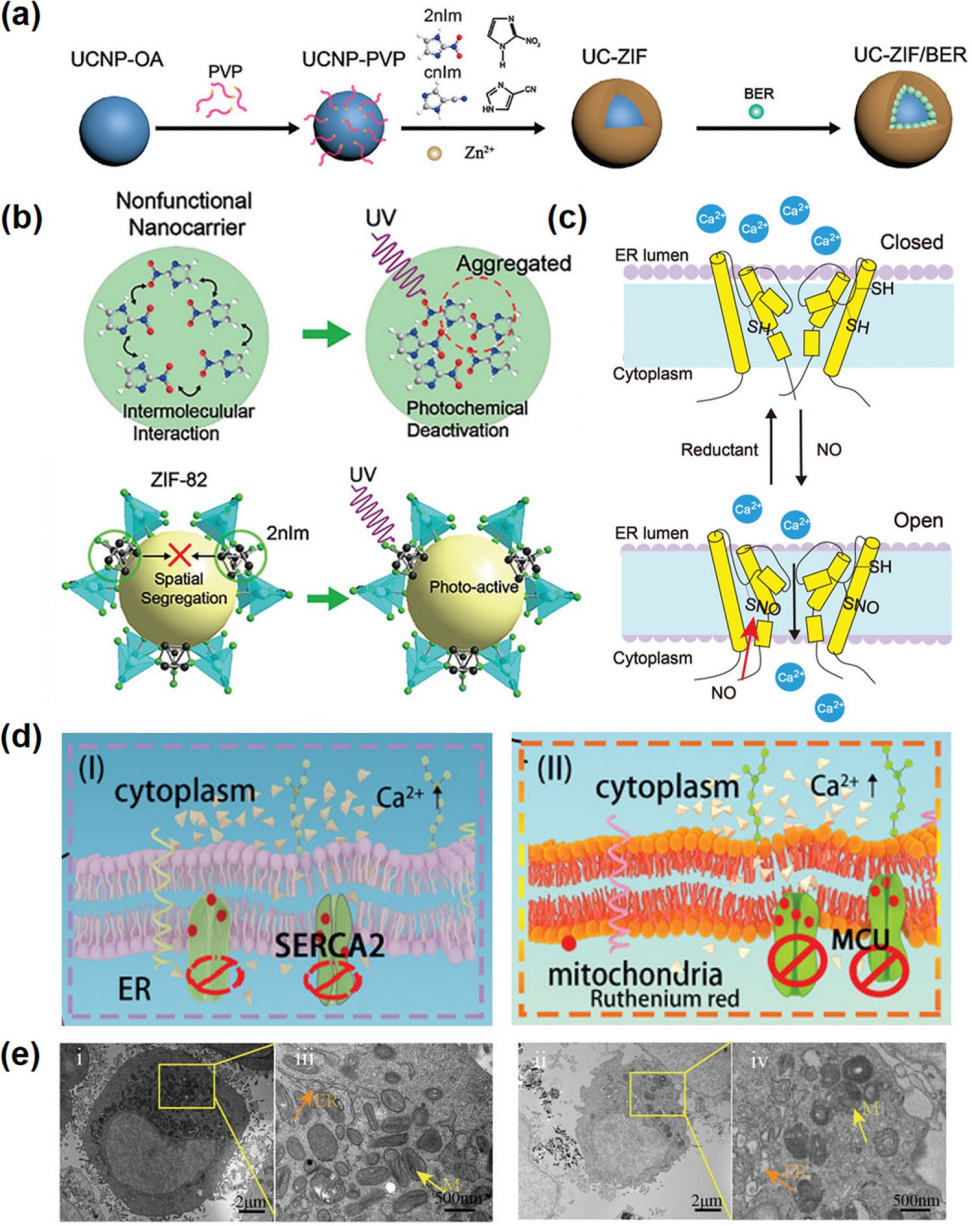
Calcium channels and calcium pumps. **a** Synthetic process of UC-ZIF/BER. **b** Aggregation quenching mechanism of 2nIm molecules in nonfunctional carrier (top panel). ZIF-82 can improve photochemical activity of 2nIm molecules (low panel). **c** Illustration of RyRS activation by NO [138]. Copyright 2021, Wiley–VCH. **d** Ca^2+^channel proteins inhibitor ruthenium red inhibited the sarcoplasmic reticulum Ca^2+^-ATPase of endoplasmic reticulum (left panel) and the mitochondrial calcium uniporter of mitochondrial (right panel) to induce calcium overload. **e** The Bio-TEM image at 0 h (left panel) and 6 h (right panel) after UCRSPH + SA-CaO_2_ NPS was incubated with 4T1 tumor cells for 5 min [73]. Copyright 2021, Wiley–VCH

The division of cancer cells is lack regulation, and the number of cell regeneration is much higher than the rate of apoptosis, in which the low cytoplasmic calcium concentration is a weapon against apoptosis of tumor cells. Studies have shown that the expression of Ca^2+^ channels/ pumps in the cell membrane decreased while the expression in the endoplasmic reticulum increased in the middle and late stage of tumor [134, 135]. This explains the continuous decrease in cytoplasmic Ca^2+^ concentration during tumorigenesis. The ubiquity of calcium signaling and its versatility within the same cell drive the evolutionary complexity of Ca^2+^ signaling, which depends on specific Ca^2+^ channels, pumps, and exchangers [136]. In fact, many Ca^2+^ pumps and channels-related activities in cancer can be modulated by specific molecular [137]. For instance, Bu et al. developed a calcium store platform by regulating calcium channels [138]. The up-conversion NPs were encapsulated with nitro-/nitrile-imidazole-based ZIF-8, which can promote the NIR triggered NO generation and the slow release of the loaded medicine (Fig. 9a–b). NO opens the overexpressed ryanodine receptors in tumor cells and suddenly elevates intracellular Ca^2+^, and berbamine (BER) closes Ca^2+^-efflux channels and prevents calcium outflow, leading to Ca^2+^ overload-mediated cell death (Fig. 9c). Such way of specifically attacking and destroying themself by regulating the calcium pumps promises to be an effective cancer treatment.

Moreover, excessive intracellular ROS levels can also affect the activity of related proteins in these calcium channels and calcium pumps. Based on the oxidation of ROS, the cysteine residues of calcium channels-related proteins are remodified, and their protein conformation and activity change accordingly. For example, strong oxidative stress can directly enhance Ca^2+^ entry into the cell through transient receptor potential (TRP) channels [139]. Meanwhile, ROS can activate TRP channels by cysteine modification, such as TRPA1 [140]. Furthermore, as a Ca^2+^ efflux pump, the function of plasma membrane Ca^2+^ ATPase (PMCA) is inhibited by ROS [141]. In general, the high intracellular ROS levels are conducive to the accumulation of cytoplasmic Ca^2+^ [142]. On this basis, Jiang et al. reported a combined drug delivery nano-platform, including UCNPsCe_6_@RuR@mSiO_2_@PL-HA (UCRSPH) and CaO_2_@HASA NPs (SA-CaO_2_) to achieve Ca^2+^ signal dysfunction [73]. CaO_2_ can simultaneously produce ROS and release calcium ions, inducing calcium channel imbalance and calcium overload. Meanwhile, RUR, a calcium channel protein blocker, synergistically restricts Ca^2+^ transport to the endoplasmic reticulum and mitochondria, controlling cytoplasm calcium levels, and ultimately leading to cell death (Fig. 9d–e). Although the potential relationship between Ca^2+^ accumulation and cell death is not quite clear, it is obvious that excessive Ca^2+^ in the cytoplasm does induce irreversible cell damage or cell apoptosis, which is most probably involved to the disruption of Ca^2+^ signaling regulation.

Calcium channel therapy leads to a new direction in cancer treatment. The intermediate conductance calcium-activated channel (IK) is involved in cancer clinical trials, and ICA-17043 is currently in phase III clinical trials as a potential drug with high potency and specificity to investigate the effects in sickle cell disease [143]. In animal studies, IK inhibitors were found to prevent the dehydration and sickle of red blood cells in mice [144]. Therefore, dysregulation of ion channels in cancers may be a potential therapeutic target and prognostic marker. And some new areas and directions that need further research, such as how calcium-based materials regulate calcium signals and the importance of calcium-based materials in ion channel-related therapy, will be the focus of future research.

### Ca-NMs for Immunotherapy

Another important therapeutic benefit of calcium-based materials is that they can enhance anti-cancer immunity [109]. Various Ca^2+^-based nanogenerators have been investigated for cancer immunotherapy. Ca^2+^ has the following advantages in cancer immunotherapy: (i) Ca^2+^ is involved in the inducing of immunogenic cell death (ICD) [43]; (ii) increased intracellular Ca^2+^ level can improve autophagy efficiency [145]; (iii) calcium-based material can be used as vaccine carrier to protect antigen activity [146]; (iv) calcium-based materials are used as immune adjuvants [147]; (v) increased intracellular calcium level can facilitate the polarization of M2 macrophages to M1 macrophages [148]. More importantly, the studies show that these Ca^2+^-based nano-modulators can effectively activate the host immune response and kill tumor cells while generating long-lasting immune memory to prevent tumor metastasis and recurrence.

#### ***ICD-Inducing Capability of Ca***^***2***+^

Tumor immunogenic cell death (ICD) promotes the production of large amounts of tumor-associated antigen (TAAs), which is an important step in the tumor immune cycle, so it has been the priority of immunotherapy for many years [149]. ICD can induce exposure calreticulin (CRT) and release high mobility group box-1 protein (HMGB-1) and adenosine triphosphate (ATP). These damage-associated molecular patterns (DAMPs) promote dendritic cells (DCs) maturation and proliferation of cytotoxic T lymphocytes, which are recognized by the immune system to activate antitumor immune responses [150]. At present, most ICD inducers are mainly chemotherapeutic drugs, such as doxorubicin (DOX), oxaliplatin (OXA), etc. [151, 152]. However, due to the limited efficiency of chemical ICD inducers, higher dosage and more frequent dosing are often required which will lead to unavoidable side effects and the drug resistance.

**Fig. 10:**
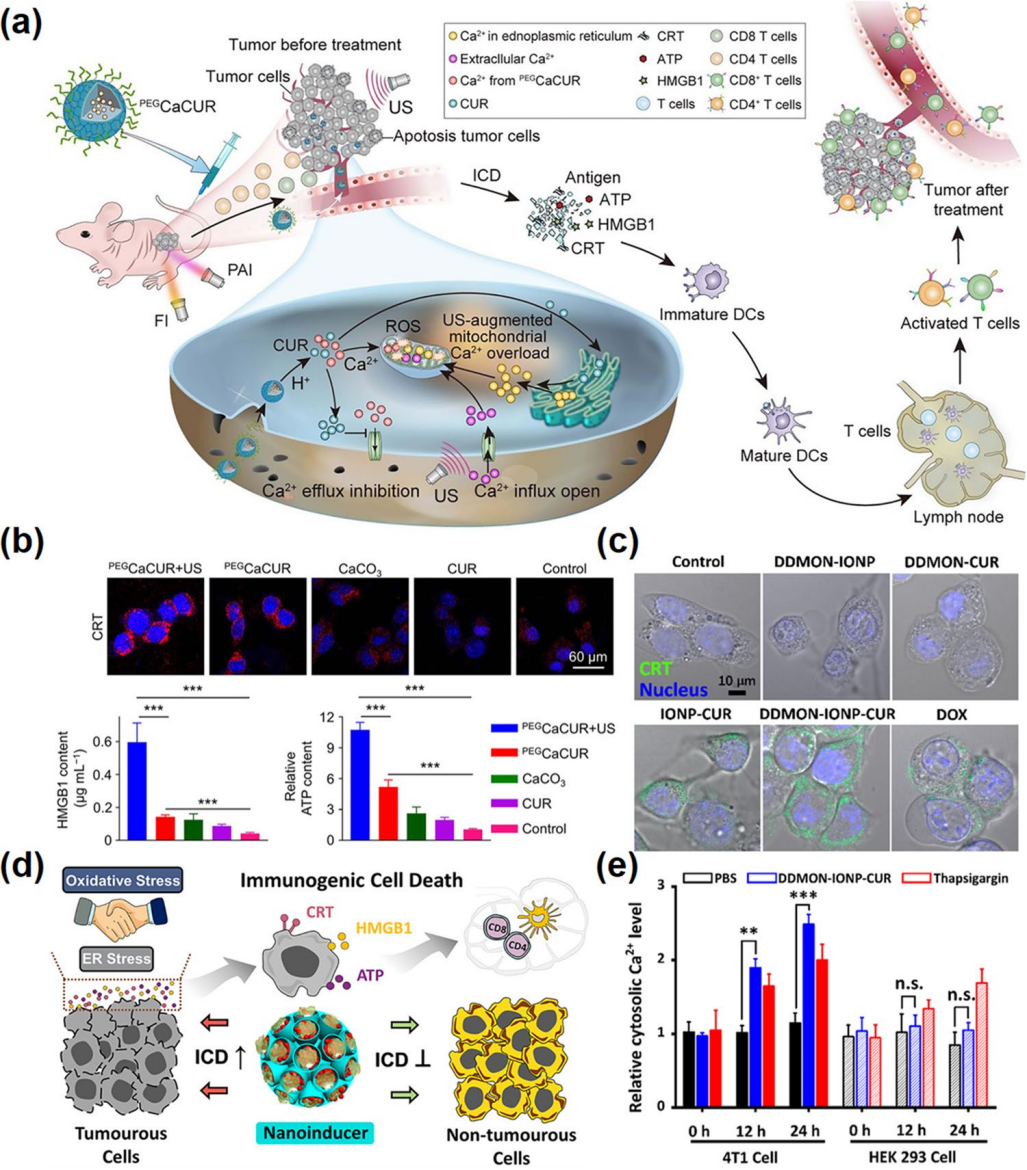
Ca-NMs-induced immunogenic cell death. **a** Schematic diagram of collaborative cancer therapy based on PA/FI imaging-guided ultrasound enhanced^PEG^ CaCUR-induced mitochondrial Ca^2+^ overload. **b** CRT exposure (top panel) and the release of HMGB1 and ATP (low panel) [43]. Copyright 2021, American Chemical Society. **c** CLSM images of CRT exposure after different treatments for 6 h. **d** Schematic illustration of ICD induced by nanoinducer. **e** The cytoplasmic Ca^2+^ level in different treatment groups [153]. Copyright 2020, American Chemical Society

Recently, Ca^2+^ has been reported as a novel ICD inducer with both efficacy and biosafety [153]. Specifically, in addition to being abundant in the cytoplasm, Ca^2+^ is also stored in organelles such as mitochondrial and endoplasmic reticulum [136]. The disruption of mitochondrial calcium homeostasis can directly regulate ROS production, stimulating DAMPs to induce ICD, and ultimately initiates defensive anti-tumor immunity. For example, Zheng et al*.* synthesized acid-sensitive polyethylene glycol (PEG)-modified CaCO_3_ nanoparticles combined with curcumin (CUR) as a multifunctional Ca^2+^ nanomodulator (PEGCaCUR) (Fig. 10a) [43]. The CaCO_3_ can directly induce the increase in overall calcium level in tumor cells. Moreover, the bioactive agents CUR can enhance mitochondrial calcium levels by promoting Ca^2+^ release from the endoplasmic reticulum (ER) to the cytoplasm and inhibiting the possible outflow. The mitochondrial Ca^2+^ overload can facilitate ROS production and induce mitochondrial damage, leading to a potent ICD effect. As shown in Fig. 10b, the PEGCaCUR can effectively evoke the release of biomarkers of ICD, including exposure of CRT, and secreta of HMGB1 and ATP. Subsequent systemic antitumor immunity was confirmed by the high levels of DCs maturation, T cells activation, and production of plentiful cytokines. Except for the disruption of mitochondrial function, previous researches have pointed that lowering the level of Ca^2+^ in the endoplasmic reticulum (ER) can also be beneficial to CRT exposure causing CRT is mostly present in the ER lumen [154]. Dai and co-workers reported a new ICD nano-inducer based on CUR by loading CUR and ferric oxide into silicon dioxide nanoparticles, which can induce ER stress by ER Ca^2+^-depleting and then effectively promote ICD (Fig. 10d) [153]. As shown in Fig. 10c, e, after nano-inducer treatment, the significant increase in cytosolic Ca^2+^ demonstrates that this drug delivery platform has a strong ER Ca^2+^-consuming ability, which is conducive to the transfer of CRT from intracellular to extracellular [154]. The immunogenic death of tumor cells ultimately promotes antigen release and presentation processes that enhance the patient's own ability to eliminate cancer cells through immune processes. Therefore, it is a reliable means to achieve effective cancer treatment based on calcium-based materials to activate and boost the patient's immune system. At the same time, immunogenic death combined with immune checkpoint inhibitors also produced better anticancer effects [155, 156].

#### ***Ca***.^***2***+^***-Mediated Autophagy***

Additionally, researches have also shown that Ca^2+^ plays an indispensable role in the autophagy process [157]. Autophagy is a smart evolutionary process of transformation of eukaryotes [158, 159]. During this process, some impaired organelles or proteins are wrapped by autophagic vacuole and transferred into lysosomes for degradation and recycling. Autophagy can facilitate the digestion and processing of antigens during antigen presentation by DCs [160, 161]. However, the autophagy ability of DCs is often inhibited in TME, which leads to a significant reduction in antigen presentation efficiency [162]. In cancer chemotherapy immunity, the effectiveness is often limited due to insufficient antigen presentation ability of DCs [163]. Thus, by inducing the maturation of DCs, effective antitumor immune responses can be activated. The homeostasis of various ions in cells controls the activities of various enzymes/proteins, such as calcium ions, which play a particularly important role in autophagy [164]. Therefore, regulation of calcium levels in DCs is possible to improve the autophagy efficiency and thus enhance immunotherapy.

**Fig. 11:**
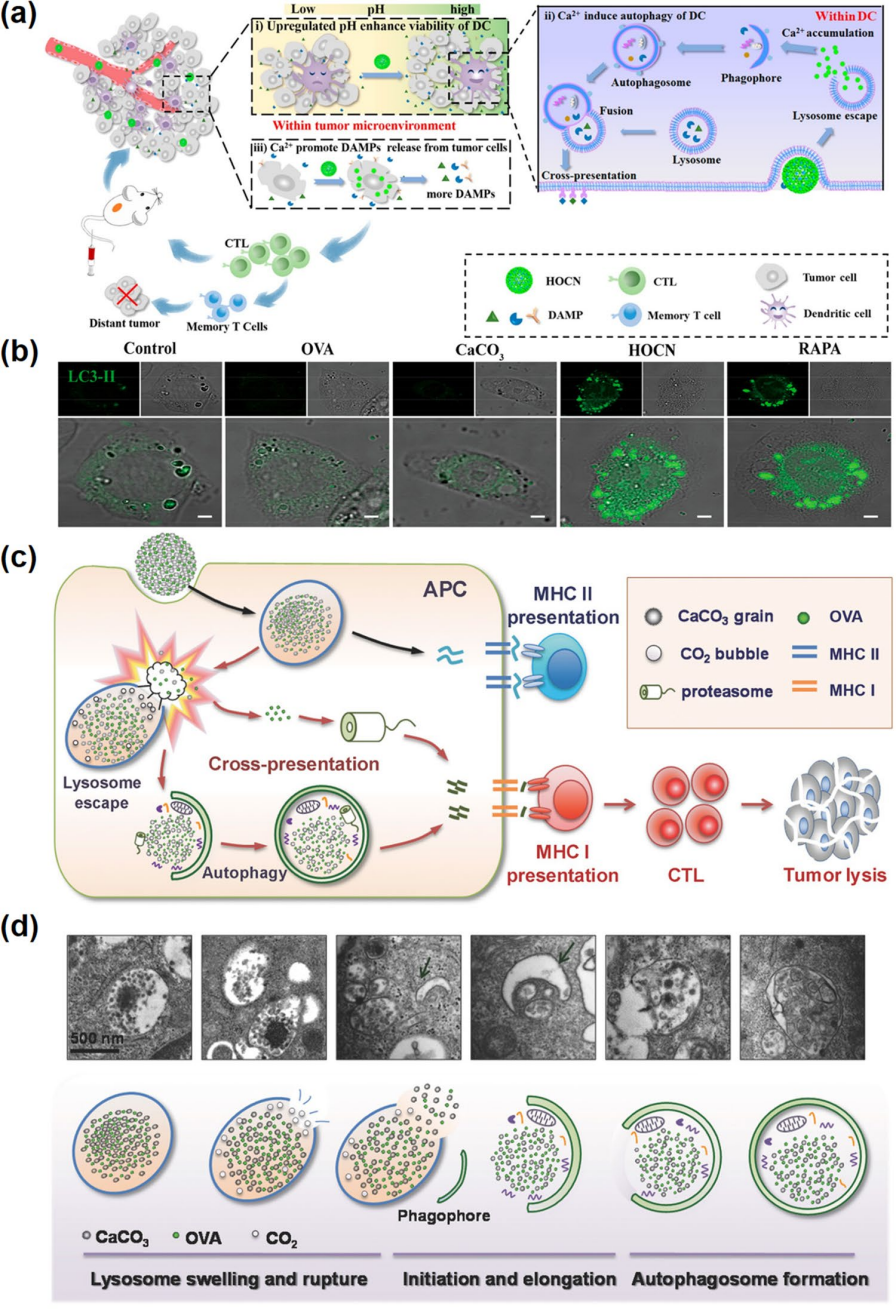
Ca-NMs-induced autophagy. **a** Schematic illustration of HOCN enhancing mitoxantrone (MTX)-mediated chemo-immunotherapy by breaking multiple barriers of DCs antigen cross-presentation. **b** Expression level of LC3-II in DCs [145]. Copyright 2020, American Chemical Society. **c** Illustration of OVA@NP-associated autophagy mediated antigenic cross presentation. **d** TEM images and relevant schemes illustration of autophagy [146]. Copyright 2018, Wiley–VCH

It has been shown that in a certain range, the autophagy ability of DCs is positively correlated with intracellular Ca^2+^ levels [165]. Therefore, increasing calcium ion levels can promote efficient antigen presentation of DCs. For example, Shi and his colleagues designed a simple Ca^2+^ nanogenerator by honeycomb CaCO_3_ loaded with a commonly used antigen OVA (HOCN), which can disrupt the multiple barriers in antigen cross-presentation within DCs, enhance DAMPs release (Fig. 11a) [145]. CaCO_3_ can also attenuate tumor acidity to alleviate the DC cell viability damage and antigen presentation ability decrease caused by TME acidity. The research reveals that this calcium ion nanogenerator showed a strong ability to overcome the barrier of antigen cross-presentation. As shown in Fig. 11b, the HOCN-induced autophagy enhancement was confirmed in DCs by LC3-II (a specific marker of autophagy). We believe that the regulation of calcium ions in tumors offers new benefits for improving cancer chemo-immunotherapy.

#### Ca-NMs as Vaccine Carriers

In other research areas, calcium-based materials also showed great potential, such as cancer vaccine design [166]. Therapeutic vaccine is one of the powerful means for tumor immunotherapy, which can improve the efficiency of the immune response and ultimately exert the therapeutic action by cytotoxic T lymphocytes (CTLs) [167]. To obtain anti-tumor cellular immunity, tumor-associated antigens must be captured by antigen-presenting cells (APCs) through major histocompatibility complex (MHC) I molecules and present them further to CD8^+^ T cells. However, in most cases, the intracellular exogenous antigens are usually decomposed by lysosomes and then guide the MHC II pathway, and evocative of CD4^+^ T cells results in subsequent humoral immunity rather than the desired cellular immunity [168]. Therefore, how to effectively deliver exogenous antigens through the MHC I pathway and further cross presentation is a scientific problem worth exploring.

Calcium-based nanomaterials have attracted broader concerns to serve as antigen protectors and carriers [41]. In particular, calcium-based carriers have been studied can avoid lysosomal degradation and promote cross presentation. For example, CaCO_3_ nanoparticles protect antigen integrity and enhance the immunogenicity of protein antigens. Based on the same material (OVA@CaCO_3_), Ma and co-workers reported that the subsequent release of CO_2_ from CaCO_3_ nanoparticles can break the lysosome membrane and achieve cytoplasmic delivery of exogenous antigens (Fig. 11c) [146]. Except for facilitating lysosome escape, the resulting burst of CO_2_ bubbles also triggers autophagy. As shown in Fig. 11d, once OVA@ CaCO_3_ NPs enter into the lysosome, the acidic environment of the lysosome leads to the rapid dissolution of CaCO_3_, accompanied by the production of large amounts of CO_2_ gas, resulting in a sharply increased pressure in the lysosome and leading to lysosome rupture. In this case, exogenous antigen OVA enters the cytoplasm and could be degraded by proteasome into epitopes peptides, and binds MHC I molecules for further cross-presentation. As a result, TME responsive calcium-based materials provide a new way to develop safe and effective therapeutic vaccines.

#### Ca-NMs as Immune Adjuvants

Adjuvants are considered as important components of effective vaccines to improve vaccine effectiveness and minimize side effects [169, 170]. The addition of adjuvants to vaccines can provide longer-lasting immune protection [171, 172]. Since Glenny et al. discovered in 1926 that diphtheria toxin (DT) suspension adsorbed by aluminum complexes had better immunogenicity than the toxoid itself, aluminum complexes have been used as vaccine adjuvants for nearly one hundred years [173]. As the first human adjuvant approved by FDA, aluminum adjuvant is still recognized as the safest and most widely used adjuvant in the world. However, it also has inherent limitations. For example, aluminum adjuvants can effectively induce humoral immunity and are difficult to stimulate the cellular immune response, while the cellular immunity is the key to tumor eradication.

To date, a variety of nanoparticles have been developed as adjuvants for vaccine systems [174, 175]. Among them, CaP NPs are considered as one of the most promising immune adjuvants [176]. Nowadays, it has been used in tetanus, diphtheria, and pertussis vaccines. Recently, the role of CaP as an adjuvant in tumor vaccine has also been paid more attention [147]. Studies have shown that CaP NPs as adjuvants can induce the immune response of helper T-cells. Meanwhile, CaP NPs have been shown to activate NLRP-3 inflammasome, thereby activating the production of cytokines such as lL-1β and co-stimulating T cell responses. Li and his colleagues designed a biomimetic antitumor nanovaccine using calcium pyrophosphate nanoparticles (CaPyro NPs) as delivery vehicles and adjuvants, and wrapping lipids and B16-OVA tumor cell membranes [147]. The preliminary results showed that CaPyro NPs accelerated the proliferation of APCs to a certain extent. Furthermore, CaPyro NPs were better engulfed by DCs, resulting in efficient antigen presentation and adaptive immune response. In another study, Peng et al. synthesized an iron and selenium-co-doped CaP nanohybrid [176]. This nanohybrid induced the rise of ROS and lead to the apoptosis of tumor cells. Meanwhile, with the combination of CaP adjuvant, the proliferation of DCs, the accumulation of CD4^+^/CD8^+^ T cells, and the expression of cytokines such as interleukin-12p70, interferon-γ (IFN-γ), and TNF-α were all observed, which effectively evoked the adaptive immune response and inhibited tumor progression. In general, CaP-based adjuvants vaccines can improve humoral and cellular immunity and enhance the immune response in populations.

#### Calcium Ions Promote Polarization of Macrophages

Macrophages are multifunctional immune cells with strong plasticity, which are engaged in a variety of physiological processes, including fighting infection, pathologic progression, and maintaining homeostasis [177, 178]. Macrophages have two main phenotypes, pro-inflammatory (M1) that helps fighting infection and (M2) that promotes anti-inflammatory and pro-healing. Notably, tumor-associated macrophages (TAMs) are the most common tumor-infiltrating immune cells, which are often presented as M2 macrophages in TME and facilitate immune escape and metastasis of tumor cells [179, 180]. Therefore, the capability of adjusting macrophage phenotypes from M2 to M1 is important for tumor immunotherapy.

**Fig. 12:**
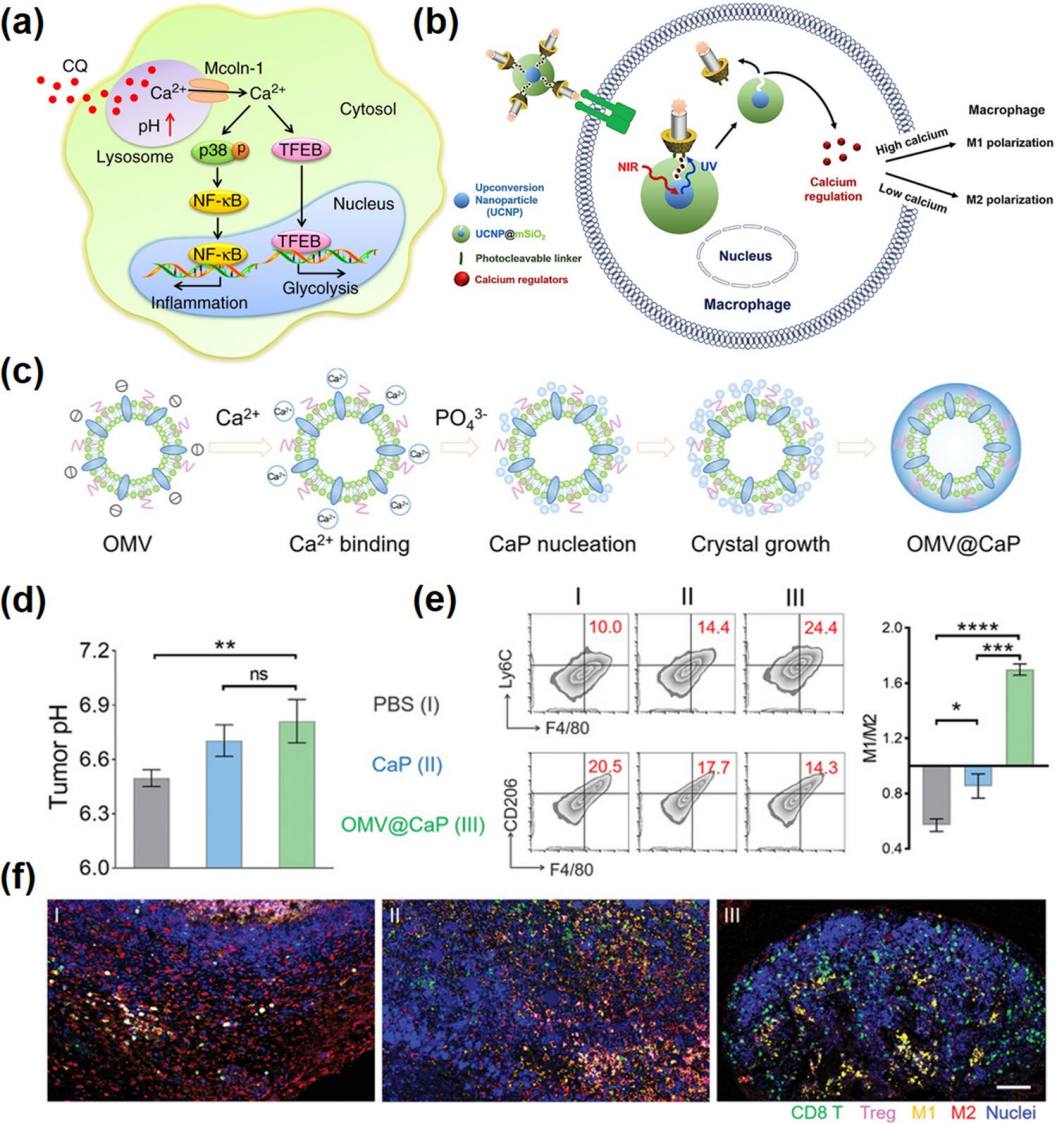
Ca-NMs promote polarization of macrophages. **a** Schematic illustration of chloroquine (CQ) resetting tumor-associated macrophages (TAMs) [183]. Copyright 2018, Springer Nature. **b** Illustration of the synthesis process of nanomaterials and experimental scheme used in this study [148]. Copyright 2018, Elsevier. **c** Scheme diagram of biomineralization procedure of OMV@CaPs. **d** Multispectral fluorescence quantification pH value of tumor tissues in different treatment groups. **e** Flow cytometry analysis of M1 macrophages and M2 macrophages, and M1/M2 ratio (right panel) in tumors. **f** Images of tumor tissue stained with Opal multicolor immunofluorescence [41]. Copyright 2020, Wiley–VCH

Macrophage polarization is regulated by a variety of signals, including chemical, mechanical, transcriptional, and chemokines [181]. There have been massive reports that confirmed the function of Ca^2+^ in regulating the polarization of macrophages, including regulating the phosphorylation of protein kinase and inhibition of M1 polarization of macrophages by Ca^2+^ channel blocking calcium outflow [182]. For example, in a chloroquine (CQ)-mediated antitumor immunity study, CQ can induce Ca^2+^ release by the calcium channel of mucolipin-1 (Mcoln1) in lysosomal; then, p38 and nuclear factor-κB (NF-κB) were activated, leading to the conversion of TAMs to the M1 phenotype (Fig. 12a) [183]. In another case, Kang et al. designed an up-conversion NPs-based nano-carrier for near-infrared (NIR) light-responsive regulate intracellular calcium signaling to reset macrophage polarization. Intracellular calcium levels were regulated by the assistance of NIR light, and the increased intracellular Ca^2+^ promoted M1-type polarization of macrophages (Fig. 12b) [148]. Similarly, the depletion of intracellular calcium inhibited the M1-type polarization but promoted the M2-type polarization of macrophages, suggesting that regulating intracellular calcium levels in macrophages is associated with their polarization phenotypes.

Calcium-based materials play an indispensable role in regulating intracellular calcium concentration. Based on this, the development of calcium-based materials for the polarization of macrophages will be a promising direction in cancer immunotherapy. In fact, various calcium-based bio-nanomaterials have been developed to control the macrophage polarization phenotypes [184]. In particular, those capabilities of sustainably releasing calcium ions show great potential in inducing the pro-inflammatory function of macrophages. For example, Ma and co-workers used CaP to encapsulate bacterial membrane vesicles (OMVs) by a “shielding” strategy (OMV@CaPs), which helped overcame the systemic inflammation of OMVs (Fig. 12c) [41]. As shown in Fig. 12d-f, the proportion of M1-type to M2-type macrophages was significantly increased in the nano-CaPs treated group, suggesting the CaPs could induce the M2–M1 polarization. Moreover, the OMV@CaPs reversed the proportion of M1 to M2, which was mainly due to the enhancement of OMV internalization by CaP and its auxiliary effect on macrophage polarization. These results revealed that calcium-based materials are involved in the reprogramming of immune cells, contributing to effective cancer treatment.

### Tumor Calcification

Biological controlled mineralization is mainly caused by the interaction between physical activities and the surrounding environment [185]. Biomineralization has unique mechanical properties and structures, and most of the time we don't quite understand how this is done step by step. Only recently have we begun to understand some of the mechanisms of mineralization and some of the problems that biological mineralization faces [53]. The deposition of ion-forming minerals in the organism is subdivided into the following steps: (i) the ions reach the biological tissue/lesion, (ii) combine with the corresponding counter-ions, (iii) both ions reach supersaturated concentration, and (iv) finally deposit as a solid phase. In reality, spontaneous calcification of certain tumors has been clinically proved to be a benign prognostic factor, such as colorectal cancer, lung cancer, and glioblastoma [106–108].

In recent years, biomineralization-inspired tumor calcification has aroused great interest in tumor therapy [186]. The tumor calcification affects the metabolism and proliferation of tumor cells, induces the metabolic disorder and dysfunction of tumor cells, and eventually leads to cell death. These calcification phenomena inspire scholars to explore and study whether Ca^2+^ enrichment in tumor foci can promote the development of tumor calcification, which has potential clinical significance for the early identification of solid tumors [107, 187]. In fact, recent researches have cleared that tumor calcification is a long, slow process of calcium mineral deposition in focal tissues caused by abnormal local accumulation of Ca^2+^ in the tumor microenvironment, which is considered to be the result of calcium overload [188, 189]. Calcification is depending on the high concentration of Ca^2+^ (above 10 mm) in the microenvironment; delivery of exogenous calcium solution into the tumor is a direct and effective method [190]. However, systemic injection of exogenous calcium can cause hypercalcemia, leading to serious side effects such as cardiac arrest, organ failure, and even death and thus cannot be used clinically.

**Fig. 13:**
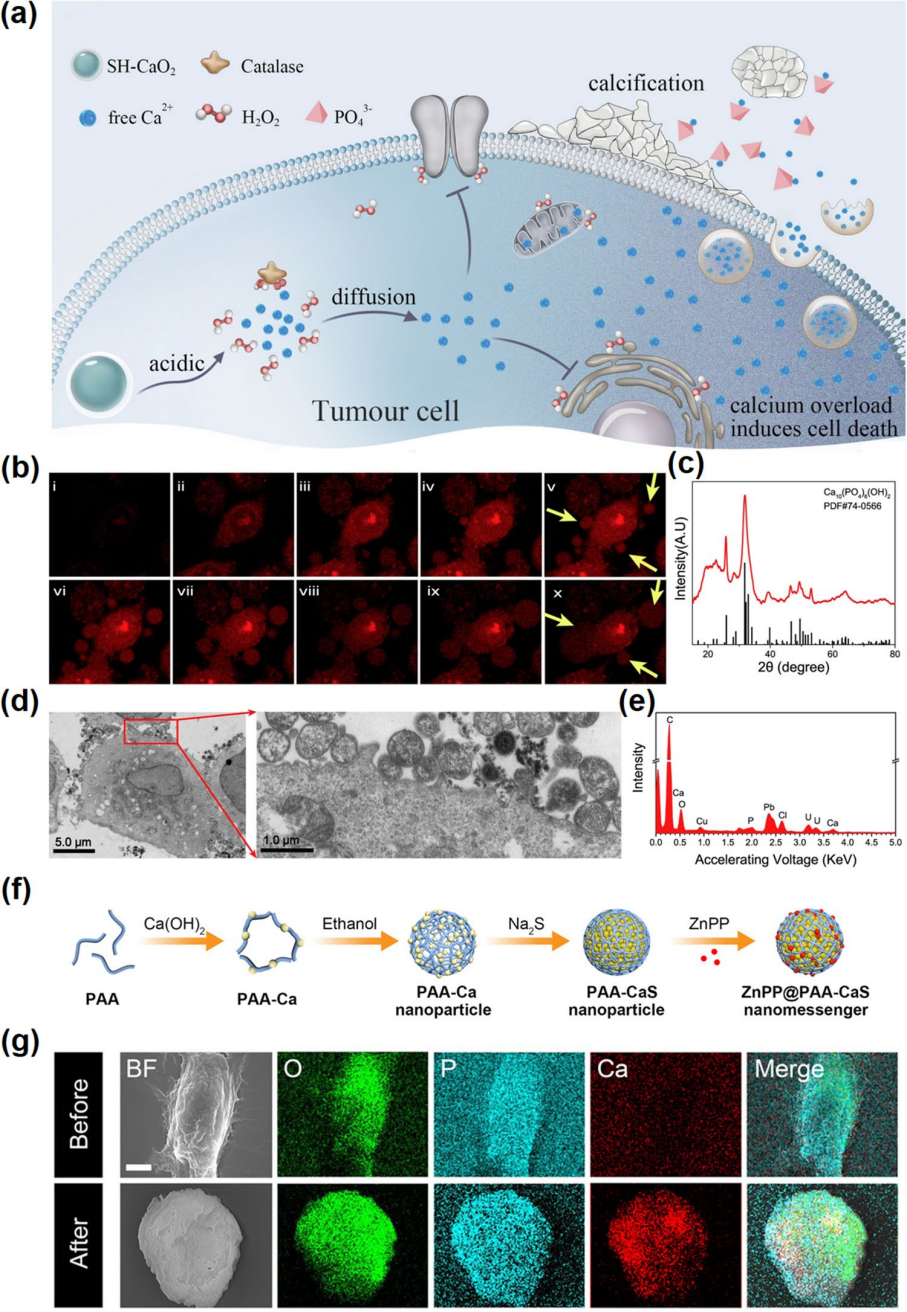
Tumor calcification. **a** Schematic illustration of the function of SH-CaO_2_ NPs in tumor cells. SH-CaO_2_ NPs are decomposed to release Ca^2+^ and H_2_O_2_ molecules in TME. Low expression of CAT in cancer cells leads to accumulation of H_2_O_2_ and imbalance of calcium transport pathways, which leads to efficient calcium overload in cells and induces cell death. Meanwhile, increased local Ca.^2+^ concentration increases the possibility of tumor calcification. **b** The yellow arrow showed calcium-enriched small vesicles formation and exocytosis after SH-CaO_2_ NPs treatment. **c** The XRD of the product collected by exocytosis of cells treated with SH-CaO_2_ NPs. **d** Bio-TEM images of calcification vesicles. **e** EDS of the extracellular products treated with SH-CaO_2_ NPs [28]. Copyright 2019, Elsevier. **f** Illustration of the synthetic procedure of a ZnPP@PAA-CaS nanomessenger. **g** Elemental mapping of 4T1 cells after ZnPP@PAA-CaS nanomessenger treatment [196]. Copyright 2021, American Chemical Society

During clinical treatment, calcification often occurs in some tumor types after chemotherapy or radiotherapy [187]. Studies have shown that the common feature of chemotherapy and radiotherapy is to produce large amounts of toxic free radicals in tumor cells [191, 192]. Although free radicals are generally thought to exert antitumor effects through directly attack on the double stranded DNA structure and causing irreversible DNA damage, cytotoxic free radical-induced dysregulation of associated calcium signaling is also thought to be the cause of cell apoptosis, which explains why therapies that produce free radicals tend to calcification [193, 194]. This indicates that induced calcium overload is not an insignificant process in the treatment, but another powerful destructive factor of disease besides free radicals. Therefore, these results suggest that the formation of calcification may involve a series of secondary reactions resulting from intracellular calcium overload induced by free radicals. For example, based on the unique biological effects of calcium overload, Bu's group modified calcium peroxide with pH-sensitive hyaluronic acid (SH-CaO_2_ NPs) [28]. To illustrate the unique cytotoxicity mechanism of calcium ions, they designed the simplest nanomaterials based on calcium peroxide to fully demonstrate the importance of calcium overload in tumor treatment (Fig. 13a). In the case of calcium overload, the cells will promote calcium ions transfer to the extracellular environment in response to calcium death. Researches have shown that calcium efflux is closely related to calcium exocytosis under intracellular calcium overload conditions [195]. At this time, cells will form and secrete a large number of “calcified vesicles” (similar to the stromal vesicles—bone-associated cells formed extracellular organelles which play a significant role in osteogenesis and biomineralization) that have the ability to absorb soluble ions in the cellular environment and precipitate them into amorphous phases, which is suspected to have a close causal relationship with cell calcification (Fig. 13b–e). As a representative work based on calcium nanomaterials, it also illustrates the great potential of calcium-based materials in clinical tumor therapy. Here, Bu et al. suggested that sodium hyaluronate (SH)-modified CaO_2_ (SH-CaO_2_) NPs have potential clinical application value in consideration of the negligible systemic toxicity, the easy accessibility of the materials, and the economy of the raw materials, exploring new ideas for the subsequent experimental research. In another research, Liu et al. developed a nanomessenger (ZnPP@PAA-CaS) to amplify the regulatory role of chemical messengers (Fig. 13f) [196]. The released chemical messengers Ca^2+^ and H_2_S can synergistically enhance intracellular Ca^2+^ stress, mediate cell death, and induce tumor calcification. As shown in Fig. 13g, scanning electron microscopy (SEM) detected significant calcium mineralization in the ZnPP@PAA-CaS-treated cells. The intracellular Ca^2+^ enrichment promotes tumor calcification, which is helpful to inhibit tumor growth, metastasis, and recurrence.

Calcification is clinically important and has been proved as a positive prognostic factor for treatment response [187]. However, tumor calcification is clinically uncommon, and the relationship between its incidence and treatment has rarely been studied until recently. Calcification reflects chronic changes in the tumor or TME over time and may be accelerated by certain treatments [187]. However, current studies do not fully explain why calcified tumors tend to show better outcomes and prognosis in late-stage of therapy. It is an important direction of a tumor therapy to explore the specific mechanism of tumor calcification and its relationship with clinical prognosis.

### Calcification-Mediated Cancer Imaging

Computed tomography (CT) scanning is sensitive to the mineralized deposits but less sensitive to cancer assessment than magnetic resonance imaging (MRI) and is therefore often overlooked in cancer monitoring [197]. Due to the specific recognition of CT to cancer calcification, it can also play a unique detection function in specific cancer types and therapeutic methods, which is helpful for medical imaging to monitor the therapeutic effect, promote the integration of diagnosis and treatment, and help clinicians judge the course of the disease [198]. The area of high attenuation (which visually resembles bone) in a CT scan abnormality is an important diagnostic clue. High attenuation on CT scan is most often caused by calcification, but can also be caused by other radiopaque foreign bodies [199]. When cancer attenuation was higher than that of the normal liver showed lower attenuation on subsequent CT scans, it was considered to be intra-cancer hemorrhage rather than calcification. The morphology of cancer calcification can be divided into spotty, ring-like, and diffuse calcification. In a clinical calcification analysis, the incidence of tumor calcification after chemoradiotherapy of single-tumor was 38.2% at 1-month CT scan and 71.4% at 6-month CT scan [187]. Spotty calcification was most common at 1-month CT scans, and diffuse calcification was most common at 6-month CT scans. Over time, the degree of tumor calcification increased, presenting a diffuse distribution. Since calcification promotes tumor remission, a diffuse pattern of tumor calcification may indicate a good tumor response. In this process, CT imaging technology provides irreplaceable technical guidance for the diagnosis and treatment of calcified tumors.

**Fig. 14:**
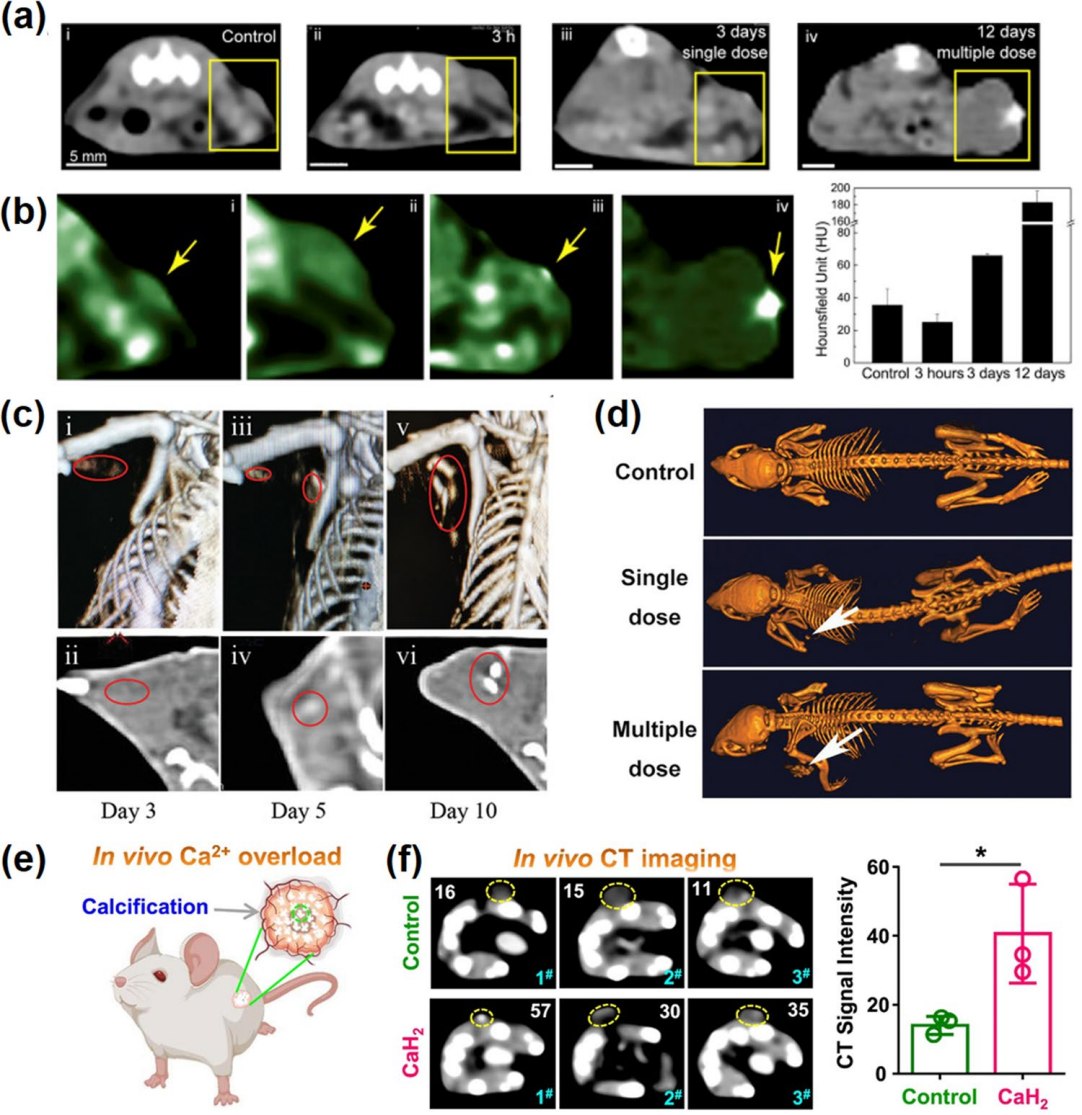
Calcification-mediated cancer imaging. **a** CT images tumors treated with SH-CaO_2_ NPs: (i) control, (ii) 3 h or (iii) 3 days after giving a single injection for a small tumor, (iv) 12 days after giving 4 doses for a big tumor. **b** Pseudocolor CT images of tumors (left panel) and their statistic [28]. Copyright 2019, Elsevier. **c** CT images at 3, 5, and 10 days after UCRSPH + SA-CaO_2_ + laser therapy [73]. Copyright 2021, Wiley–VCH. **d** CT images of mice in the control group, single dose group and multiple dose group after treatment [188]. Copyright 2021, Wiley–VCH. **e** Schematic diagram showing calcification induced by calcium overload after injection of nano-CaH_2_. **f** CT images of nano-CaH_2_-injected and the individual Hounsfield unit (HU) values [92]. Copyright 2022, Elsevier

It has been clinically proved that tumor calcification can be considered a benign prognostic indicator after therapy [107, 187]. Therefore, based on the CT imaging to tumor calcification, the researchers developed a series of combination diagnoses and therapy. For instance, the above-mentioned SH-CaO_2_ NPs presented a good example. In addition to playing a good therapeutic role, it can also accelerate the formation of tumor calcification, providing visualization. As shown in Fig. 14a and b, 3 days after a single injection, the tumor area on CT image became significantly brighter, and microcalcification became more obvious after multiple injections [28]. In another similar case, Jiang and co-workers reported a CaO_2_-based nanoparticles to disturb Ca^2+^ signal and enhance PDT [73]. Dense microcalcification was occurred in the tumor region five days after treatment, and the CT signal was enhanced after 10 days (Fig. 14c). Moreover, Liu et al. developed a combined therapy involved in H_2_S gas, enzyme dynamic therapy (EDT), and Ca^2+^-interference therapy [188]. As shown in Fig. 14d, a, clear CT signal appeared at the tumor region after 3 days of treatment. And the signal was further strengthened after 14 days, indicating the CT signal was enhanced with the enhancement of calcification with the extension of treatment, supervising the whole process of therapeutic. In addition to traditional drug delivery methods, intervention or intra-tumoral injection can directly deliver drugs to the tumor site, increasing the concentration of drugs at the target site. For example, Liu et al*.* prepared nano-CaH_2_, which can react with H_2_O to produce Ca^2+^, hydrogen (H_2_), and hydroxyl ions (OH^−^), accelerating tumor calcification (Fig. 14e–f) [92]. After injection of nano-CaH_2_, CT signal of the tumor was significantly enhanced due to the sharp increase in Ca^2+^ concentration in the tumor. The progress of calcification was well visualized by CT imaging, which can in turn promote the CT signals to monitoring the therapeutic process. In future studies, cancer calcification will be used as an integral part of theranostics and an important indicator of improved prognosis, urging the development of more clinically oriented calcification-imaging based treatment systems.

## Main Challenges of Ca-NMs

Mounting studies are developing various Ca^2+^ nanogenerators for cancer treatment, while the current Ca-NMs still have limitations. The ideal Ca^2+^ nanogenerator should have the advantages of easy preparation, low toxicity, high drug efficiency, and promising clinical transformation prospects, which is still a huge task. While paying attention to calcium-related nano-therapeutics, the main challenges also cannot be ignored.

### Therapeutic Efficiency of Ca-NMs

Therapeutic efficacy is the original intention of all drug delivery systems. Due to the unique therapeutic mechanism, calcium has both advantages and limitations in treatment effect. Among them, the treatment short board hinders the function of the agents, seriously reduced the overall efficacy [200]. Due to the calcium present in the body with considerable levels, it is difficult to reach effective therapeutic concentrations [10]. Moreover, the powerful regulation function of Ca^2+^ channels/pumps leads to the rapid recovery of Ca^2+^ overload to normal concentration, resulting in a poor anti-cancer efficacy. In addition, the ubiquity of calcium signaling makes it difficult to identify precise therapeutic targets. In other words, Ca-NMs may damage normal cells when they inhibit calcium signaling [11]. Thus, specific substrates that can only be activated within the TME need to be developed.

### Biosafety of Ca-NMs

In recent years, the role of calcium in cancer treatment has been gradually highlighted due to the calcium overload, calcification, calcium immunity, etc. bringing multilevel therapeutic function. Although calcium-based materials have shown encouraging performance in cancer therapy, their specific biosafety issues in vitro and in vivo are not well understood. Calcium is an element contained in the human body, which has good biocompatibility and biodegradability. However, when the content of calcium exceeds the limit, the effect of an overloaded calcium homeostasis regulation system is not quite clear. At the same time, the inevitable drug leakage in body fluid circulation is a long-lived issues of nano-drug delivery system. In particular, excessive calcium ion leakage into blood vessels will cause hypercalcemia and thrombosis [201]. In fact, most studies have routinely described systemic toxicity or immune responses after injecting Ca-NMs into mice, which is clearly insufficient for systematic biosafety assessment and needs to be further explored in future researches.

### Clinical Translation of Ca-NMs

Ca-NMs have clear advantages and are an attractive drug delivery system in the biomedical field; however, they also have some disadvantages or limitations that hinder their clinical application. Firstly, as the drug carriers, their drug delivery capacity is limited compared with other polymer drug delivery systems [202]. In this case, the drug concentration is difficult to reach the clinically effective concentration, which greatly affects the overall clinical efficacy. Secondly, the instability of the preparation process is another serious defect, and the uncontrollable reaction speed will result in the formation of large particles, hindering the efficiency of cell internalization. And this kind of uncontrollability is more significant in the expansion of pharmaceutical scale, because it is difficult to achieve large-scale production of drugs. Moreover, although Ca-NMs have well-known pH-accelerated release properties, their release kinetics and exact release behavior under normal conditions have not been studied in detail, and uncontrolled slow release has been observed under normal physiological conditions, which will cause unknown effects on normal tissues. Finally and most importantly, the potential threats and risks of nanoparticles have received increasing attention. In addition to the inherent untargeted retention caused by different nano-sizes [203, 204], further studies are needed to investigate the adverse effects of calcium homeostasis disorders in non-target sites due to the non-specific release of Ca^2+^.

## Conclusion and Prospects

As many of the examples outlined above attest, the calcium ions are an ingenious tool to regulate key calcium-related tumor mechanisms. As summarized in Table 1, we highlighted the therapeutic mechanisms of calcium-related materials for cancer therapy, including disequilibrium of calcium homeostasis, calcium overload stress, “calcicoptosis,” ion interference strategies, calcium channels/pumps dysfunction, calcium-related immunotherapy, tumor calcification, and calcification-related CT imaging. The antitumor effect and calcium signaling are not only unidirectional promotion but also mutually reinforcing or synergistic in tumor therapy, which provided a new perspective on cancer, and efforts to target calcium signaling could change the therapeutic model of cancer.

**Table 1:** A Summary of Major Application and Functionalities of Ca-NMs

Ca-based NMs	Ca Type	Role	Functionalities	References
SH-CaO_2_ NPs	CaO_2_	Therapeutic agent	Ca^2+^ overload, tumor calcification, CT imaging	[28]
AIM NPs	CaCO_3_	Carrier/therapeutic agent	Control release, neutralize tumor acidity	[32]
BSO-TCPP-Fe@CaCO_3_-PEG	CaCO_3_	Carrier/therapeutic agent	Control release, Ca^2+^ overload,	[33]
^PEG^CaNM_CUR+CDDP_	CaCO_3_	Carrier/therapeutic agent	Control release, Ca^2+^ overload	[34]
DOX/HAP-HA	HAp	Drug carrier/ therapeutic agent	Control release, induce mitochondrial damage in tumor cells	[35]
HA NPs	HAp	Therapeutic agent	Amplified oxidative stress, Ca^2+^ overload,	[36]
GOx-MnCaP-DOX	CaP	Carrier	Control release	[40]
OMV@CaPs	CaP	Carrier/therapeutic agent	Control release, neutralize tumor acidity	[41]
M@CaCO_3_@KAE NPs	CaCO_3_	Carrier/therapeutic agent	Ca^2+^ overload	[42]
^PEG^CaCUR	CaCO_3_	Therapeutic agent	Mitochondrial Ca^2+^ overload, induce ICD	[43]
DNCaNPs	CaCO_3_	Carrier/therapeutic agent	Control release, neutralize tumor acidity	[44]
DOX-CaCO_3_-MNPs	CaCO_3_	Drug carrier	Ultrasound imaging	[45]
HCLO NPs	CaCO_3_	Drug carrier	Control release	[46]
mPEG–PEI–AuNRs & mPEG-PEI/CaNPs	CaCO_3_	Therapeutic agent	Photoacoustic imaging	[50]
CaCO_3_@COF-BODIPY-2I@GAG	CaCO_3_	Therapeutic agent	Ca^2+^ overload	[51]
Alg-CaCO_3_-PDA-PGED	CaCO_3_	Gene carrier	Gene delivery, ultrasound imaging	[54]
NMOF@DHA@CaCO_3_	CaCO_3_	Drug carrier/ therapeutic agent	Control release, Ca^2+^- mediated oncosis therapy	[55]
aCD47@CaCO_3_	CaCO_3_	Carrier/therapeutic agent	Control release, proton scavenger	[57]
LCP-CD/ICG-BsAb NPs	CaP	Gene carrier	Control release	[61]
GMCD	CaP	Drug carrier	Control release	[68]
TiO_2_@CaP	CaP	Carrier/therapeutic agent	Control release, Ca^2+^ overload	[69]
Fn@CaP	CaP	Carrier/therapeutic agent	Control release, neutralize tumor acidity, immunomodulation, tumor calcification	[70]
CaO_2_/Cu–ferrocene	CaO_2_	Therapeutic agent	Ca^2+^ overload, amplified oxidative stress	[71]
CaO_2_ − CuO_2_@HA NC	CaO_2_	Therapeutic agent	Ca^2+^ overload, tumor calcification, CT imaging	[72]
SA-CaO_2_	CaO_2_	Therapeutic agent	Ca^2+^ overload, tumor calcification, neutralize tumor acidity, CT imaging, produce O_2_	[73]
CaO_2_@ZIF-8/DOX@HA	CaO_2_	Drug carrier/therapeutic agent	Control release, neutralize tumor acidity, mitochondrial Ca^2+^ overload, tumor calcification, CT imaging	[74]
CaO_2_-Cu/ICG@PCM NPs	CaO_2_	Therapeutic agent	Neutralize tumor acidity, Ca^2+^ overload, tumor calcification, CT imaging	[75]
CaO_2_@DOX@ZIF-67	CaO_2_	Therapeutic agent	Produce O_2_ and H_2_O_2_	[78]
(MSNs@CaO_2_-ICG)@LA NPs	CaO_2_	Therapeutic agent	Amplified oxidative stress	[79]
pZIF-8/pHA-G scaffold	HAp	Bone scaffold	Bone tissue regeneration, osteoconductivity	[86]
HA-DOX@MSNs/HAP & oHA-DOX@MSNs/HAP	HAp	Carrier	Control release	[87]
Fe-CaSiO_3_ composite Scaffolds	CaSiO_3_	Bone scaffold/carrier/therapeutic agent	Enhance the degradation of scaffold, promote osteogenic differentiation	[90]
nano-CaH_2_	CaH_2_	Therapeutic agent	Ca^2+^ overload, neutralize tumor acidity, hydrogen therapy, immunomodulation	[92]
HCaM-PB	CaSiO_3_	Carrier	Control release	[95]
CaF_2_:Eu NPs	CaF_2_	Carrier, scintillator	Ca^2+^-induced radiosensitization	[98]
CaNP@cAD-PEG	CaO_2_	Therapeutic agent	“Ca^2+^ interference” induced reset M2-like TAMs to M1 phenotype, induce ICD	[109]
CaBPs	CaP	Carrier/therapeutic agent	Control release, Ca^2+^ overload, changing the osmotic pressure	[119]
OVA@CaCO_3_	CaCO_3_	Therapeutic agent	Neutralize tumor acidity, induce autophagy of DCs, promote DAMPs release	[145]
OVA@NP	CaCO_3_	Antigen carrier/therapeutic agent	Promote lysosome escape-mediated antigen cross-presentation, induce autophagy	[146]
CM@CaPyro NGs	CaPyro	Vaccine carrier/ immunoadjuvant	Control release, increase the delivery and uptake efficiency of antigens, induce Th1 and Th2 based immune responses,	[147]
OVA/CaCO_3_/PLY	CaCO_3_	Antigen carrier	Control release, enhance the immunogenicity of protein, promote lysosome escape-mediated antigen cross-presentation	[166]
Fe/Se–CaP	CaP	Carrier/immunoadjuvant	Improve DCs accumulation, boost adaptive immune response	[176]
DCC-HA NCs	CaO_2_	Therapeutic agent	Ca^2+^ overload, tumor calcification, CT imaging	[188]
ZnPP@PAA-CaS	CaS	Therapeutic agent	Ca^2+^ overload, tumor calcification, induce ICD	[196]

Although promising advances have been made in calcium-related tumor therapy, further research is needed in this field, where there is room for improvement. More consummate calcium tumor therapy should also consider the following points thoroughly.

1. Ion interference is an advanced therapeutic concept, but all ion pathways need to be subject to the chemical limits of ion operation and the “manager” role of ions in many cellular functions. Ion interference alone may not achieve the ideal therapeutic effect since the appearance of multiple Ca^2+^ transport channels in cancer cells regulates intracellular Ca^2+^ homeostasis, and cancer cells show a superior ability to adapt Ca^2+^ interference, often resulting in ineffective tumor therapy. Moreover, whether this ion interference therapy system can be used to treat drug-resistant tumor cells remains unclear. Could this cascade of changes in cellular calcium overload introduce other risks? There are still many questions to be explored.
2. Calcium plays a significant role in regulating innate immune sensing and host defense against invading pathogens. The discovery of the multiple immune functions of Ca^2+^ and understanding the mechanisms of action in immune regulation are still in the primary stage, and most reports just stay on a proof of concept. The detailed mechanism of action is not very clear, and many of them are only proven in vitro, resulting in unmet needs for clinical transformation. It is advisable to actively explain the theoretical mechanism of calcium immunity and pay attention to its clinical effects.
3. Tumor calcification is an important supporting factor for calcium-based materials to embody their clinical value. However, the authentic and exact reason behind calcification inducing a good prognosis still needs further exploration. Artificially guiding tumor calcification and providing accurate treatment are important in the clinic. In addition, the occurrence of tumor calcification not only requires excessive Ca^2+^ but also phosphate anions are the indispensable conditions for calcium mineral formation. Exploring an appropriate proportion of calcium and phosphorus content design system to obtain the most appropriate and non-toxic ion concentration is a vital task for calcium therapy.
4. More information on the underlying mechanism is needed to establish causation relationships and to further link calcium-dependent targets and pathways to calcium therapeutic. In particular, the presence of intracellular basal calcium levels leads to insensitivity to monitoring changes in calcium signaling. Much of the data generated by ion channels and subcellular organelle techniques are often difficult to interpret. Thus, new calcium signaling chemical probes must be constantly developed, specifically at the subcellular resolution level. The relationship between calcium and cancer requires further research in cancer biology, including tumor initiation, growth, and metastasis. Advanced sequencing, proteomics, metabolomics, and other advanced analytical methods should be applied to explore new biological information about calcium and cancer (or other diseases). Focus on the clinical guidance of drugs, promote drugs regression clinical value, and reinterpreting the principles and mechanisms in disease evolution should be the direction of efforts.

Overall, developing calcium-based materials for specific cancer therapy is a recognized trend. This system involves many disciplines, including materials science, chemistry, molecular biology, medicine, and imageological; a more in-depth interdisciplinary study addressing calcium-based materials for specific cancer therapy is now necessary. With the development of the calcium-cancer relationship and the further understanding of cancer, we believe that more efficient and multifunctional calcium-based delivery systems will be designed to enhance therapy efficiency and facilitate clinical transformation. Furthermore, we believe that calcium-based materials will continue to be responsible for breakthroughs in cancer treatment and expect it to become a paragon of a new generation of anticancer agents.
